# MESPBO: Multi-Strategy-Enhanced Student Psychology-Based Optimization Algorithm for Global Optimization Problems and Feature Selection Problems

**DOI:** 10.3390/biomimetics11010037

**Published:** 2026-01-05

**Authors:** Guolin Zhai, Sai Li

**Affiliations:** 1 College of Tourism, Resources and Environment, Zaozhuang University, Zaozhuang 277160, China; zhaiguolin@uzz.edu.cn; 2 School of Foreign Languages, Qufu Normal University, Jining 273165, China; 3 College of Mechanical and Electrical Engineering, Zaozhuang University, Zaozhuang 277160, China; 4 State Key Laboratory of Infrared Detection, Shanghai Institute of Technical Physics, Chinese Academy of Sciences, Shanghai 200083, China

**Keywords:** feature selection, student psychology-based optimization algorithm, meta-heuristic algorithm, global optimization problems

## Abstract

Feature selection and continuous optimization are fundamental yet challenging tasks in machine learning and engineering design. To address premature convergence and insufficient population diversity in Student Psychology-Based Optimization (SPBO), this paper proposes a Multi-Strategy-Enhanced Student Psychology-Based Optimizer (MESPBO). The proposed method incorporates three complementary strategies: (i) a hybrid heuristic initialization scheme based on Latin Hypercube Sampling and Gaussian perturbation; (ii) an adaptive dual-learning position update mechanism to dynamically balance exploration and exploitation; (iii) a hybrid opposition-based reflective boundary control strategy to enhance search stability. Extensive experiments on the CEC2017 benchmark suite with 10, 30, and 50 dimensions demonstrate that MESPBO consistently outperforms 11 state-of-the-art metaheuristic algorithms. Specifically, MESPBO achieves the best Friedman mean ranks of 2.00, 1.67, and 1.67 under 10D, 30D, and 50D settings, respectively, indicating superior convergence accuracy, robustness, and scalability. In real-world feature selection tasks conducted on 10 benchmark datasets, MESPBO achieves the highest average classification accuracy on 9 datasets, reaching 100% accuracy on several datasets, while maintaining competitive performance on the remaining one. Moreover, MESPBO selects the smallest feature subsets on 7 datasets, typically retaining only 2–4 features without sacrificing classification accuracy. Compared with the original SPBO, MESPBO further reduces the fitness values on 7 out of 10 datasets, achieving an average improvement of approximately 10%. These results verify that MESPBO provides an effective trade-off between optimization accuracy and feature compactness, demonstrating strong adaptability and generalization capability for both global optimization and feature selection problems.

## 1. Introduction

With the rapid integration of big data and artificial intelligence, machine learning has become an essential analytical tool across diverse fields, including medical diagnosis [1], financial risk assessment [2], image processing [3], and natural language understanding [4]. The effectiveness of these models, however, heavily depends on the quality and relevance of their input features. In real-world datasets, it is common for the feature space to contain redundant, irrelevant, or noisy attributes, which can obscure the intrinsic patterns of data. The presence of such redundant information not only amplifies computational complexity and prolongs training time but also triggers the curse of dimensionality, making optimization in high-dimensional spaces extremely challenging. More critically, it increases the likelihood of overfitting, where a model performs well on the training set but fails to generalize to unseen data [5]. To mitigate these issues, feature selection (FS) has emerged as a fundamental step in data preprocessing. The core objective of FS is to identify a compact subset of informative and discriminative features that preserves the essential characteristics of the original dataset while eliminating redundancy and noise. By reducing dimensionality, feature selection not only enhances learning efficiency and model interpretability but also improves classification accuracy and generalization capability [6]. Consequently, it has become an indispensable component of modern machine learning pipelines and plays a pivotal role in constructing efficient, reliable, and explainable intelligent systems.

Despite the significance of feature selection, identifying the optimal subset of features from high-dimensional data remains an NP-hard combinatorial optimization problem. Traditional methods—such as filter [7], wrapper [8], and embedded approaches [9]—often suffer from limited search capability or dependency on specific learning models, which restricts their generalization across diverse datasets. In particular, filter methods rely heavily on statistical correlations and may overlook complex nonlinear dependencies among features, while wrapper and embedded methods are computationally expensive and prone to overfitting when dealing with large-scale datasets.

In recent years, intelligent optimization algorithms inspired by nature and social behavior have garnered significant attention due to their powerful global search capabilities and flexibility in handling complex, nonlinear, and multimodal search spaces [10]. These algorithms, known as meta-heuristic algorithms, have been successfully applied in fields such as engineering design [11], machine learning, energy management [12], and feature selection [13]. For instance, Mozhdehi et al. proposed a novel Sacred Religion Algorithm based on an evolutionary socioeconomic approach inspired by religious societies [14]. This algorithm models interactions among followers, missionaries, and leaders, demonstrating outstanding performance across 23 standard benchmark functions and five practical optimization problems. Liu et al. proposed a novel graduate student evolutionary algorithm inspired by the daily behaviors of graduate students [15]. By simulating key processes such as identifying research directions and focusing on studies, the algorithm established a mathematical model for GSEA. It demonstrated favorable results on the CEC2017 and CEC2022 test sets. Furthermore, it exhibited the capability to solve real-world optimization problems in unmanned aerial vehicle and robot path planning tasks. Fu et al. simulated the search, pursuit, predation, and food storage behaviors of the Eurasian magpie to establish a mathematical model of RBMO [16]. This model demonstrated remarkable performance across the CEC2014 (Dim = 10, 30, 50, and 100), CEC2017 (Dim = 10, 30, 50, and 100) suites, drone path planning, and five engineering design problems.

Furthermore, as the no free lunch (NFL) theorem demonstrates, no single optimization algorithm performs exceptionally well across all problems. When averaged across all optimization problems, all algorithms exhibit identical performance. This implies that we should select algorithms based on the specific problems we need to solve. For instance, Tang et al. proposed a multi-strategy particle swarm optimization hybrid dandelion optimization algorithm to address the issues of slow optimization speed and susceptibility to local optima in the dandelion optimization algorithm. This approach was developed specifically for three engineering design problems of varying complexity that required resolution. Experimental results demonstrated that the algorithm achieved significant improvements in solving these three problems [17]. To address deployment challenges in wireless sensor networks, Bao et al. proposed a multi-strategy integrated group teaching optimization algorithm (MSIGTOA) employing strategies such as chaotic inverse learning. This approach achieved higher coverage while reducing node usage by at least 10%, thereby significantly lowering WSN deployment costs [18]. To address the issue of intraday operation optimization in microgrids, Lu et al. developed an Enhanced Sardine Optimization Algorithm (ESOA) incorporating composite adversarial learning. Through validation in microgrid dispatch applications, it demonstrated significant improvements over the standard Sardine Optimization Algorithm (SOA) [19].

Among various metaheuristics, the Student Psychology-Based Optimization (SPBO) algorithm, inspired by the learning behavior and psychology of students in a classroom, has recently shown promising performance in balancing exploration and exploitation [20]. SPBO models the process of students learning from top-performing peers and improving their knowledge based on collective interaction and self-learning. Despite its advantages, the original SPBO still suffers from limitations such as random initialization, fixed learning parameters, and inefficient boundary handling, which can hinder its convergence stability and search performance in complex optimization landscapes.

Many researchers have improved SPBO to better address the optimization problems they need to solve. For example, to address power system optimization problems, Balu et al. proposed a novel quasi-oppositional chaotic student psychological optimization algorithm [21]. In two radial distribution systems, considering different load models under three load levels, the algorithm achieves optimal location and size for distributed generation and parallel capacitors, yielding excellent results. To perform big data clustering, Shanmugam et al. proposed a robust and effective IoT routing technique based on SPBO [22]. By performing feature selection during the mapping stage, they effectively improved clustering performance in terms of energy, clustering accuracy, Jaccard coefficient, and Land coefficient. To address the economic dispatch problem, Basu et al. proposed an improved SPBO [23]. Experiments on economic dispatch problems involving valve point results, limited feasible area, ramp rate boundaries, and multi-pipe fueling demonstrate that the MSPBO is capable of providing better results.

To overcome these challenges, this paper proposes a Multi-Strategy-Enhanced Student Psychology-Based Optimization (MESPBO) algorithm. The proposed method integrates several improvement strategies to enhance the robustness, adaptability, and convergence efficiency of the original SPBO. Specifically, a hybrid heuristic population initialization mechanism based on Latin Hypercube Sampling (LHS) and Gaussian perturbation is introduced to improve the diversity and uniformity of the initial population. Furthermore, an adaptive dual-learning position update mechanism dynamically adjusts the learning intensity and direction of each individual according to iteration progress and population diversity, ensuring a smooth transition from exploration to exploitation. Additionally, a hybrid opposition-based reflective boundary control strategy is designed to prevent the loss of potentially valuable individuals and maintain population diversity near the boundaries.

To evaluate the effectiveness of MESPBO, extensive experiments are conducted on a set of well-known benchmark functions from the IEEE CEC2017 test suite, as well as on feature selection problems from real-world datasets. Comparative analyses with 11 state-of-the-art algorithms demonstrate that MESPBO achieves superior optimization accuracy, faster convergence speed, and better robustness across different problem categories. Moreover, the proposed algorithm shows strong generalization capability, making it suitable for both continuous global optimization and discrete feature selection tasks.

The main contributions of this work are summarized as follows:

1. A hybrid heuristic initialization strategy combining LHS and Gaussian perturbation is proposed to ensure a well-distributed and diverse initial population.
2. An adaptive dual-learning mechanism is developed to dynamically balance exploration and exploitation throughout the optimization process.
3. Introduce hybrid oppositional reflection boundary control to enhance the stability and diversity of population evolution and improve the boundary control performance of the algorithm.
4. Comprehensive experiments on benchmark and real-world datasets validate the superior performance and general applicability of MESPBO.
5. The effectiveness of the MESPBO algorithm in solving practical problems was comprehensively analyzed by applying it to the practical application of photovoltaic model parameter extraction.

The remainder of this paper is organized as follows: Section 2 reviews the student psychology-based optimization algorithm. Section 3 presents the detailed formulation of the proposed MESPBO algorithm and its improvement strategies. Section 4 provides experimental settings and performance analysis on benchmark functions. Section 5 discusses the results and comparisons on feature selection tasks, and finally, Section 6 concludes the paper and outlines future research directions.

## 2. Student Psychology-Based Optimization Algorithm

Since the MESPBO proposed in this paper is an improvement upon SPBO, this section provides a brief introduction to SPBO. SPBO was conceived through research into student behavior across different schools and colleges, drawing inspiration from insights into student psychology. Bikash Das et al. categorize students into four groups: top performers, good students, average students, and those randomly attempting to improve. Each category exhibits distinct psychological activities, which are used to model the algorithm’s iterative update process. Specific details are as follows:

### 2.1. Best Student

Typically, the student who achieves the highest score on an exam is regarded as the top student. To maintain this position, the top student consistently strives to earn the highest grade in the class, necessitating greater effort. Consequently, the top student’s effort process can be modeled as shown in Equation (1).

(1) $$X_{bestnew}=X_{best}+{\left(-1\right)}^{k}\times rand\times \left(X_{best}-X_{j}\right),$$

where $X_{best}$ and $X_{j}$ represent the top student and the j-th randomly selected student in a specific subject, respectively. rand denotes a random number between 0 and 1, while k is a parameter randomly selected as either 1 or 2.

### 2.2. Good Student

If a student develops an interest in any subject, they will attempt to invest increasing effort into that subject to enhance their overall performance. Such students are defined as good students. The choices made by these students constitute a random process due to variations in student psychology. To achieve the highest scores on exams and become the best students, some students strive to exert effort comparable to or exceeding that of the top performers. The specific effort process of such students can be modeled by Equation (2).

(2) $$X_{newi}=X_{best}+\left[rand\times \left(X_{best}-X_{i}\right)\right],$$

where $X_{i}$ denotes the i-th top student. Additionally, some students exert greater effort in their studies than their peers in the class and strive to emulate the efforts of the most accomplished students. The effort process of such students can be modeled by Equation (3).

(3) $$X_{newi}=X_{i}+\left[rand\times \left(X_{best}-X_{i}\right)\right]+\left[rand\times \left(X_{i}-X_{mean}\right)\right],$$

where $X_{mean}$ indicates the class’s average performance in a specific subject.

### 2.3. Average Student

Since the effort students exert depends on their interest in the subjects offered to them, if students are less interested in certain subjects, they will exert average effort in those subjects to improve their overall grades. Such students are defined as average students. Given the differing psychological profiles of students, their choices also constitute a random process, which can be modeled by Equation (4).

(4) $$X_{newi}=X_{i}+\left[rand\times \left(X_{mean}-X_{i}\right)\right],$$

### 2.4. Students Who Try to Improve Randomly

**Figure 1:**
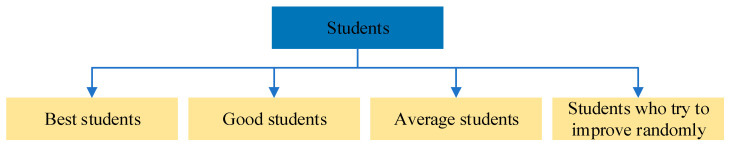
Student classification diagram.

**Figure 2:**
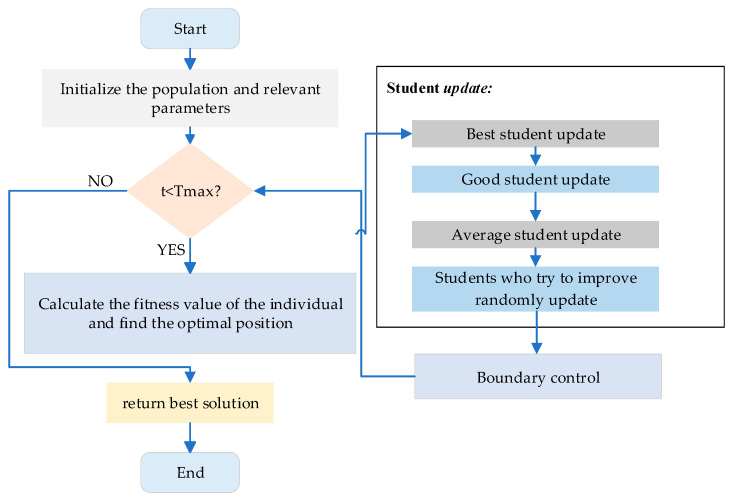
The flowchart of SPBO.

In addition to the aforementioned three categories of students, some students attempt to improve their grades independently. They strive to enhance their overall exam performance by applying effort somewhat randomly across subjects. The efforts of this group of students can be specifically modeled as Equation (5).

(5) $$X_{newi}=X_{min}+\left[rand\times \left(X_{max}-X_{min}\right)\right],$$

where $X_{max}$ and $X_{min}$ represent the upper and lower bounds of the problems to be solved, respectively, and also denote the maximum and minimum scores achievable in the student subjects. The classification of students is shown in Figure 1. Algorithm 1 presents the pseudocode for the SPBO algorithm. Figure 2 presents the flowchart of the SPBO algorithm.
**Algorithm 1:** the pseudo-code of the SPBO1: ***Begin*** 2: Initialize the relevant parameters and the population 3: ** *while*** $t<T_{max}$ 4:    Evaluate the initial performance of the class 5:    check the category of the student 6: ***   Best students:*** 7:      Modify performance by Equation (1) 8: ***   Good students:*** 9:      Modify performance by Equations (2) and (3) 10: ***   Average students:*** 11:      Modify performance by Equation (4) 12: ***   Students who try to improve randomly:*** 13:      Modify performance by Equation (5) 14:    Check the boundary 15:    Update the students’ performance 16: *** End while*** 17: *** return*** best student 18: ***end***

## 3. Proposed MESPBO

Although the SPBO algorithm demonstrates good optimization capability inspired by student psychological behaviors, its search performance can still be limited by insufficient population diversity, static learning mechanisms, and inefficient boundary control. To overcome these drawbacks, the Multi-strategy-Enhanced Student Psychology-Based Optimization (MESPBO) introduces three major improvements, focusing on the population initialization, student position updating, and boundary control mechanisms.

### 3.1. Hybrid Heuristic Population Initialization

In the original SPBO, the initial population is generated randomly, which may lead to uneven distribution and weak exploration ability in the early stage. To enhance the population diversity and improve convergence performance, MESPBO introduces a hybrid heuristic initialization mechanism, which integrates Latin Hypercube Sampling (LHS) [24] and Gaussian perturbation [25].

#### 3.1.1. Latin Hypercube Sampling (LHS)

For a D-dimensional optimization problem, the initial population matrix is denoted by Equation (6).

(6) $$X=[x_{i,j}{]}_{N\times D},\;i=1,2,\ldots ,N;j=1,2,\ldots ,D$$

where N denotes the population size, while D represents the problem’s dimensionality. The j-th dimension is divided into N equally spaced intervals, which can be expressed by Equation (7).

(7) $$I_{k}^{\left(j\right)}=\left[\frac{k-1}{N},\frac{k}{N}\right],k=1,2,\ldots ,N.$$

Then, one sample is randomly selected from each interval and permuted to ensure non-overlapping coverage, it can be expressed by Equation (8).

(8) $$x_{i,j}^{LHS}=L_{j}+\left(U_{j}-L_{j}\right)\cdot \pi_{j}\left(r_{i}\right),$$

where $L_{j}$ and $U_{j}$ are the lower and upper bounds of the j-th dimension, $r_{i}\in I_{k}^{\left(j\right)}$ is a uniformly distributed random number in the k-th interval, and $\pi_{j}(\cdot )$ is a random permutation function ensuring each interval is used once. This process guarantees that all samples are uniformly distributed in the search space.

#### 3.1.2. Gaussian Perturbation

To prevent clustering and enhance local search diversity, Gaussian noise is applied to each generated solution. Specifically, it can be expressed as Equation (9).

(9) $$x_{i,j}^{G}=x_{i,j}^{LHS}+N\left(0,\sigma_{j}^{2}\right),$$

where $N(0,\sigma_{j}^{2})$ is a Gaussian perturbation with zero mean and variance $\sigma_{j}^{2}$, and it can be expressed as Equation (10).

(10) $$\sigma_{j}=\kappa \cdot \left(U_{j}-L_{j}\right),$$

where κ∈[0.01,0.05] is a control parameter determining perturbation intensity. In summary, the final initial population can be expressed as Equation (11).

(11) $$X=X^{LHS}+N\left(0,\Sigma \right),$$

where $\Sigma =diag(\sigma_{1}^{2},\sigma_{2}^{2},\ldots ,\sigma_{D}^{2})$.

In the initialization stage, each individual is first generated by Latin Hypercube Sampling to ensure global uniformity. Then, a Gaussian perturbation is applied to each dimension of the population to introduce local randomness, thereby enhancing population diversity and preventing premature clustering in specific regions.

This hybrid initialization mechanism combines the global uniformity of Latin Hypercube Sampling with the local stochasticity of Gaussian perturbation. It ensures a well-distributed and diverse initial population, which effectively improves global exploration and convergence stability.

### 3.2. Adaptive Dual-Learning Position Update Mechanism

In the original SPBO, each category of students updates their positions using fixed coefficients, resulting in a rigid exploration–exploitation balance that may not adapt to different optimization stages. To overcome this limitation, the proposed MESPBO introduces an adaptive dual-learning mechanism, where students dynamically adjust their learning intensity according to both iteration progress and population diversity [26].

Adaptive Learning Coefficients: At the beginning of the optimization, maintaining high population diversity is crucial for avoiding local optima; hence, a stronger global exploration component is adopted. As iterations proceed, the algorithm gradually shifts its focus toward local exploitation to refine the solutions around the best individuals. This transition is controlled by two time-varying learning coefficients α(t) and β(t). The coefficients α(t) and β(t) decrease smoothly with the number of iterations, can be expressed as Equations (12) and (13)

(12) $$\alpha (t)=\alpha_{max}-(\alpha_{max}-\alpha_{min})\times {\left(\frac{t}{T}\right)}^{\lambda },$$

(13) $$\beta (t)=\beta_{min}+(\beta_{max}-\beta_{min})\times \left(1-e^{-\mu t/T}\right),$$

where t is the current iteration count; T is the maximum iteration count; $\alpha_{max}$ and $\alpha_{min}$ control the exploration range; $\beta_{max}$ and $\beta_{min}$ control the exploitation range; and λ and μ are adaptation factors controlling the decay rate. The values of λ and μ are both set to 1.2, allowing the algorithm to explore better in the early stages and develop better in the later stages. α(t) dominates global exploration in the early stage, while β(t) gradually strengthens local exploitation in later iterations.

Dual-Learning Position Update Formula: The position of the $i^{th}$ student is updated according to both the best individual and the population mean, it can be expressed by Equation (14).

(14) $$X_{i}^{new}=X_{i}+\alpha (t)\cdot rand_{1}\cdot (X_{best}-X_{i})+\beta (t)\cdot rand_{2}\cdot (X_{i}-X_{mean}),$$

where $rand_{1}$ and $rand_{2}$ represent random numbers between 0 and 1, $X_{best}$ indicates the global optimum, and $X_{mean}$ denotes the average value across the entire population.

The adaptive dual-learning strategy enables each student to dynamically balance between exploration and exploitation according to the optimization phase and population diversity. Early in the search, larger α(t) values promote exploration of the global search space. As the iteration progresses, smaller α(t) and larger β(t) values focus the search around promising regions, thus improving convergence accuracy and stability.

### 3.3. Hybrid Opposition-Based Reflective Boundary Control

Boundary handling is critical for preserving the stability and continuity of the population evolution. Instead of the common truncation or random re-initialization, MESPBO adopts a hybrid opposition-based and reflective boundary control [27]. When an individual component $X_{i,d}$ goes outside its feasible interval $\left[X_{min,d},X_{max,d}\right]$, it is remapped by either a reflective mapping or an opposition mapping. This hybrid strategy helps to keep infeasible individuals in the search process, reintroduce diverse candidate solutions, and reduce the likelihood of getting stuck in local optima.

Reflective mapping: When individuals in the algorithm exceed the bounds, reflective mapping can be used to reflect the out-of-bounds individuals back into the feasible domain, which can be specifically expressed as Equation (15).

(15) $$X_{i,d}^{ref}=\left\{\begin{array}{c} X_{min,d}+\left|X_{i,d}-X_{min,d}\right|,\;\;X_{i,d}<X_{min,d}\\ X_{max,d}-\left|X_{i,d}-X_{max,d}\right|,\;\;X_{i,d}>X_{min,d} \end{array}\right..$$

Opposition mapping: The opposition mapping assigns outlier individuals to opposing positions within the interval to enhance the algorithm’s exploration capability, which can be calculated using Equation (16).

(16) $$X_{i,d}^{opp}=X_{min,d}+X_{max,d}-X_{i,d}.$$

Additionally, to enhance the algorithm’s ability to escape local optima, we introduce small perturbations to opposing particles to increase diversity, which can be expressed as in Equation (17).

(17) $$X_{i,d}^{opp,\epsilon }=clip\left(X_{i,d}^{opp}+\epsilon ,X_{min,d},X_{max,d}\right),$$

where clip(⋅,a,b) restricts the value to [a,b].

Hybrid strategy: When individual $X_{i,d}$ crosses the boundary, it selects the opposition mapping with probability P, otherwise it selects the reflection mapping. This can be expressed as Equation (18).

(18) $$X_{i,d}^{new}=\left\{\begin{array}{c} clip\left(X_{i,d}^{opp}+\epsilon_{1},X_{min,d},X_{max,d}\right),\;\;with\;probability\;P\\ clip\left(X_{i,d}^{ref}+\epsilon_{2},X_{min,d},X_{max,d}\right),\;\;with\;probability\;1-P \end{array}\right.,$$

where $\epsilon_{1}$ and $\epsilon_{2}$ represent random disturbances following a uniform distribution.

**Figure 3:**
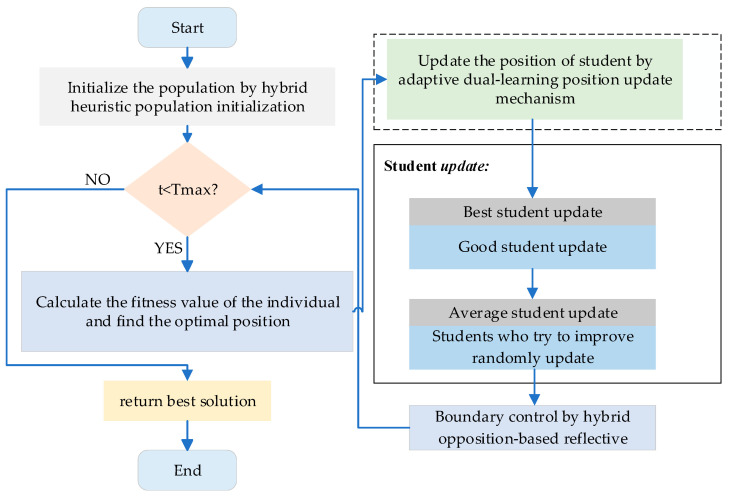
The flowchart of MESPBO.

To robustly handle out-of-bounds individuals, we propose a hybrid opposition-based reflective boundary control. When a decision variable exceeds its feasible range, it is remapped either by an opposition mapping $X_{opp}=X_{min}+X_{max}-X$ or by a reflective mapping that mirrors the violation back into the feasible interval. The choice between opposition and reflection is governed by a probability P. Small random perturbations are applied to the remapped values to avoid deterministic cycles, and the final result is clipped to $[X_{min},X_{max}].$ This hybrid mechanism preserves search continuity, reintroduces potentially promising candidates, and enhances population diversity, thereby reducing the likelihood of premature convergence. To visually illustrate the algorithm’s execution process, Figure 3 presents the flowchart of the MESPBO algorithm.

### 3.4. Time Complexity Analysis

Time complexity analysis is essential for any heuristic algorithm, as it directly reflects the algorithm’s scalability and computational efficiency on large-scale problems. In this section, we analyze the time complexity of MESPBO, where the main computational overhead comes from the iterative loop and the update operations for all individual students in each iteration. Specifically, the algorithm needs to traverse all students in each iteration and execute an update strategy based on their category. Therefore, the overall time complexity of the algorithm can be expressed as O(N×T), where N is the population size and T is the maximum number of iterations. The time complexity is the same as the original SPBO algorithm, without affecting the algorithm’s performance by orders of magnitude. In conclusion, the improvement to SPBO is acceptable in terms of time complexity.

## 4. Experimental Analysis of Global Optimization Problems

### 4.1. IEEE CEC2017 Benchmark Suite

To comprehensively evaluate the performance of the proposed MESPBO algorithm, the IEEE CEC2017 benchmark suite is employed as the standard test platform. The CEC2017 test set is a well-established and widely recognized benchmark collection designed by the IEEE Congress on Evolutionary Computation for assessing the performance of real-parameter optimization algorithms [28]. It consists of 30 continuous optimization functions, including unimodal, multimodal, hybrid, and composition functions, which progressively increase in complexity. These functions effectively represent different optimization challenges, such as local optima entrapment, high-dimensional nonlinearity, and strong variable interactions.

The diversity and difficulty of the CEC2017 test suite make it an authoritative benchmark for verifying the global search capability, convergence accuracy, robustness, and stability of intelligent optimization algorithms. Moreover, it provides a fair and unified testing environment that facilitates direct comparison with other state-of-the-art algorithms. Therefore, this paper adopts the CEC2017 benchmark suite to rigorously test the proposed MESPBO algorithm, ensuring that its performance evaluation is objective, comprehensive, and consistent with current research standards in the optimization community.

### 4.2. Comparison of Algorithms and Parameter Settings

In this section, the effectiveness of the proposed MESPBO algorithm is systematically examined on the widely recognized CEC2017 benchmark suite and benchmarked against multiple competitive optimization algorithms. The comparison algorithms include: Particle Swarm Optimization (PSO) [29], Snake Optimization (SO) [30], Gold Rush Optimizer (GRO) [31], Secretary Bird Optimization Algorithm (SBOA) [32], enterprise development optimization algorithm (ED) [33], Escape optimization algorithm (ESC) [34], hyper-heuristic whale optimization algorithm (HHWOA) [35], Improved Grey Wolf Optimizer (IGWO) [36], Modified Student Psychology-Based Optimization algorithm (MSPBO) [23], Quasi-oppositional chaotic student psychology-based optimization algorithm (QOCSPBO) [21], and Student Psychology-based optimization algorithm (SPBO) [20]. The algorithms parameters are listed in Table 1. All experiments were conducted in a Windows 11 environment using an AMD Ryzen 7 9700X octa-core processor (3.80 GHz) with 48 GB of memory and MATLAB 2024b software.

### 4.3. Experimental Results and Analysis of CEC2017 Test Suite

**Figure 4:**
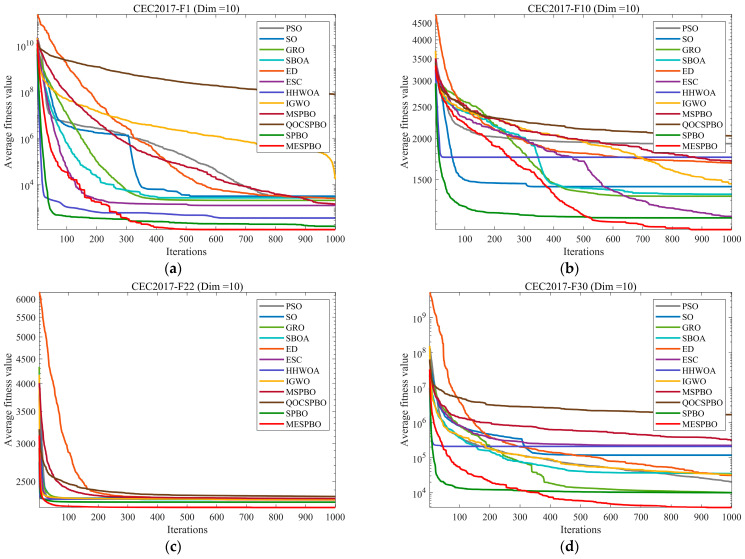

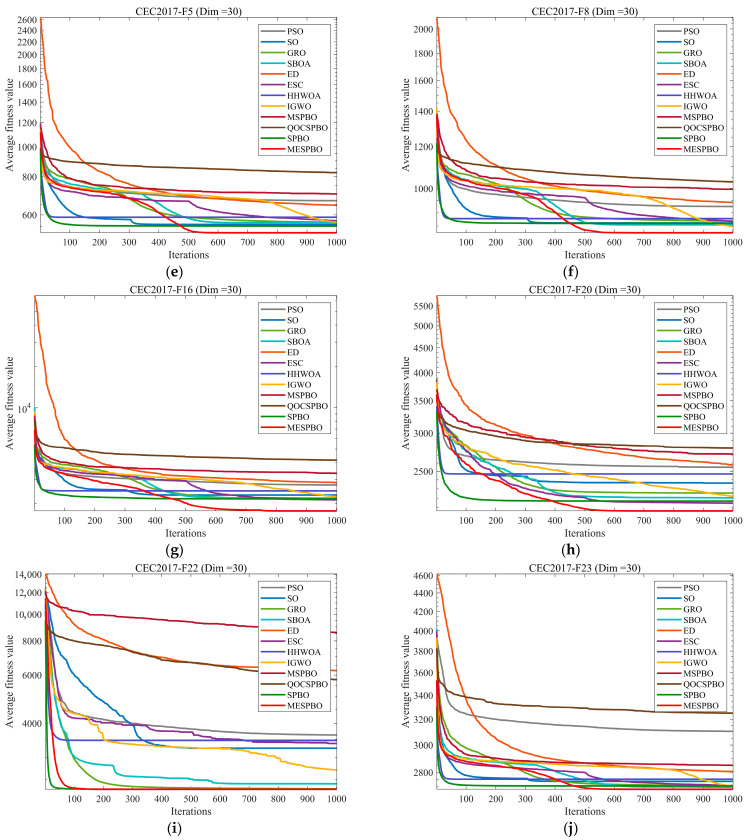

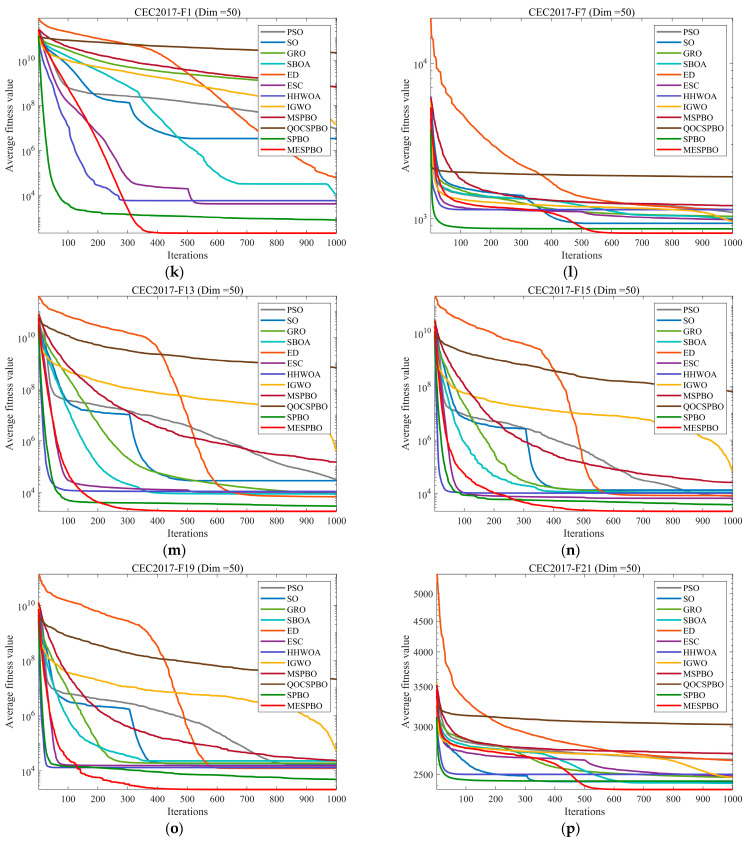
Comparison of convergence speed of different algorithms on CEC2017 test set. (**a**) cec2017-f1 (dim = 10), (**b**) cec2017-f10 (dim = 10), (**c**) cec2017-22 (dim = 10), (**d**) cec2017-30 (dim = 10), (**e**) cec2017-f5 (dim = 30), (**f**) cec2017-f8 (dim = 30), (**g**) cec2017-f16 (dim = 30), (**h**) cec2017-f20 (dim = 30), (**i**) cec2017-f22 (dim = 30), (**j**) cec2017-f23 (dim = 30), (**k**) cec2017-f1 (dim = 50), (**l**) cec2017-f7 (dim = 50), (**m**) cec2017-f13 (dim = 50), (**n**) cec2017-f15 (dim = 50), (**o**) cec2017-f19 (dim = 50), (**p**) cec2017-f21 (dim = 50).

**Figure 5:**
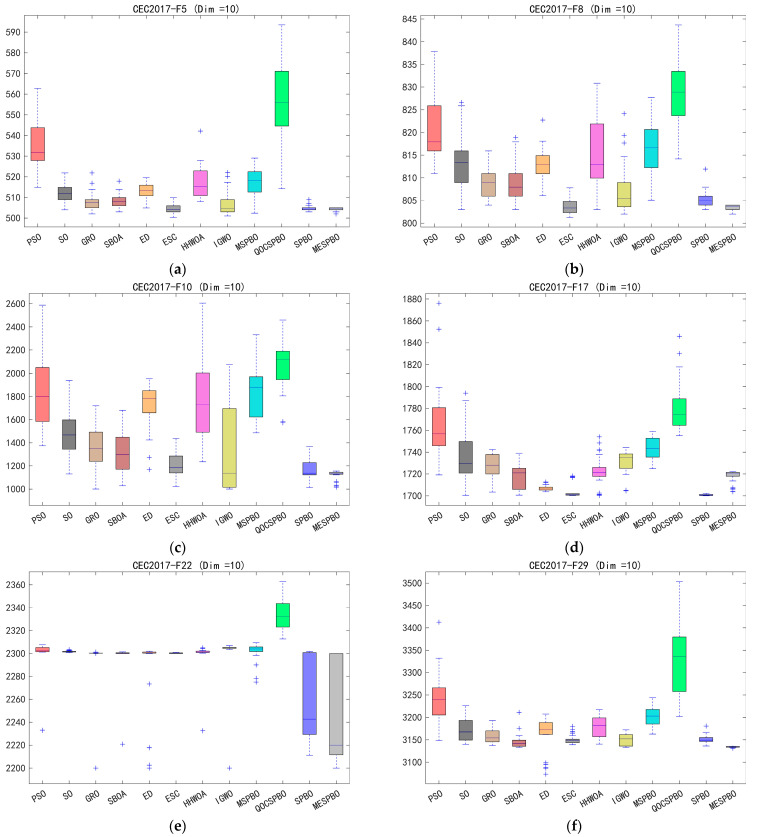

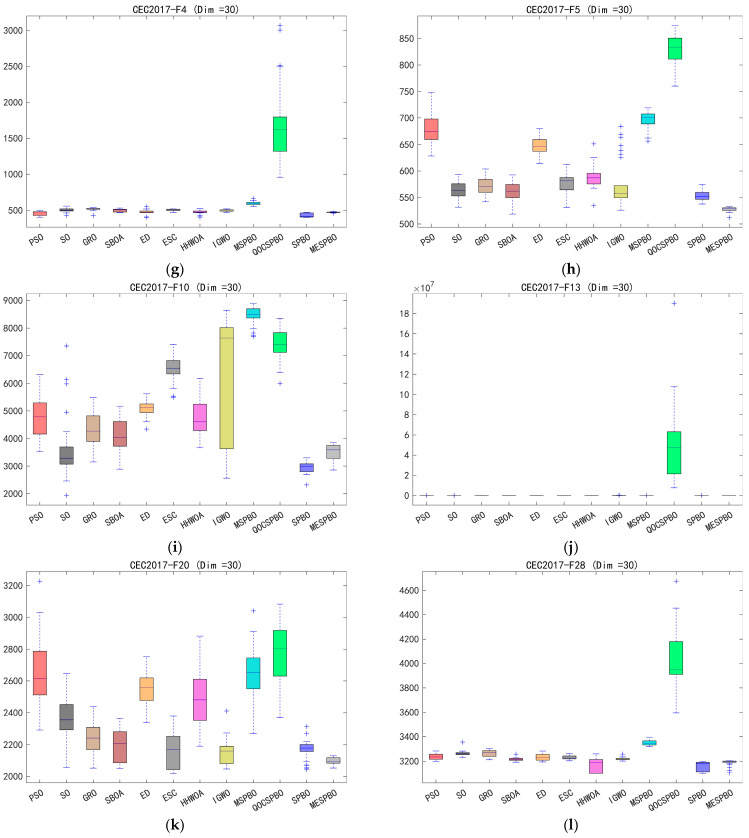

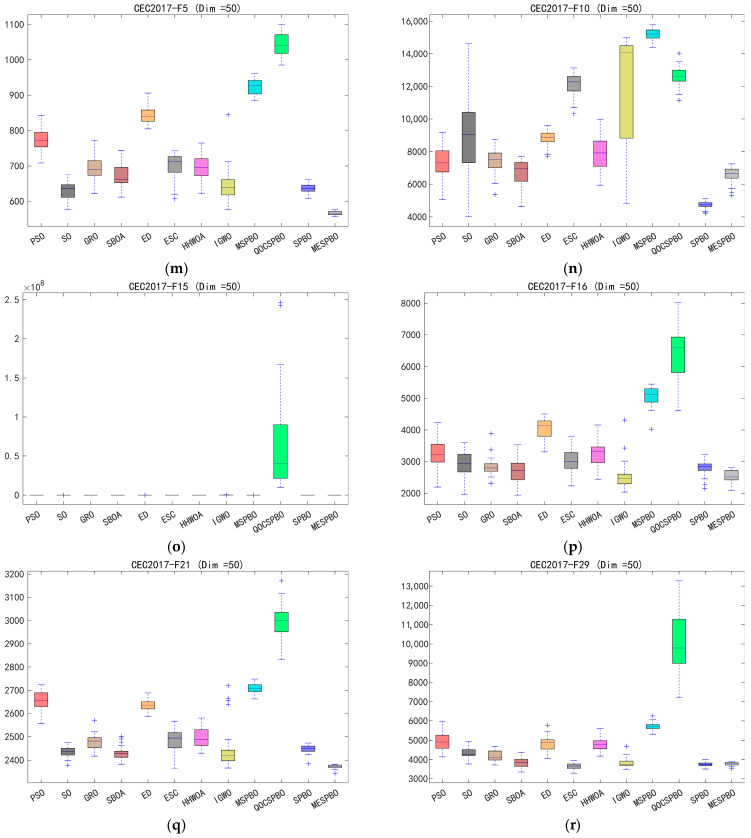
Boxplot analysis for different algorithms on the CEC2017 test set. (**a**) cec2017-f5 (dim = 10), (**b**) cec2017-f8 (dim = 10), (**c**) cec2017-10 (dim = 10), (**d**) cec2017-17 (dim = 10), (**e**) cec2017-f22 (dim = 10), (**f**) cec2017-f29 (dim = 10), (**g**) cec2017-f4 (dim = 30), (**h**) cec2017-f5 (dim = 30), (**i**) cec2017-f10 (dim = 30), (**j**) cec2017-f13 (dim = 30), (**k**) cec2017-f20 (dim = 30), (**l**) cec2017-f28 (dim = 30), (**m**) cec2017-f5 (dim = 50), (**n**) cec2017-f10 (dim = 50), (**o**) cec2017-f15 (dim = 50), (**p**) cec2017-f16 (dim = 50), (**q**) cec2017-f21 (dim = 50), (**r**) cec2017-f29 (dim = 50).

This section evaluates the performance of MESPBO using the CEC2017 benchmark suite. To comprehensively test its capabilities, experiments were conducted on CEC2017 functions with dimensions of 10, 30, and 50. To ensure fairness, the population size for all algorithms was set to 50, and the maximum number of iterations was set to 100. To mitigate the impact of algorithmic randomness on results, each algorithm was independently run 30 times. Table 2, Table 3 and Table 4 records the mean (Ave) and standard deviation (Std) from these 30 independent runs. For a more intuitive analysis of the experimental outcomes, Figure 4 presents the convergence curves of the algorithms. To comprehensively display the results from the 30 runs, Figure 5 shows the box plots of the algorithms.

The convergence curves on the CEC2017 benchmark under 10D, 30D, and 50D scenarios indicate that MESPBO achieves the best overall performance on most test functions. Specifically, MESPBO exhibits a markedly faster decrease in average fitness than PSO, SO, GRO, SBOA, ED, ESC, HHWOA, IGWO, MSPBO, QOCSPBO, and SPBO, while continuing to refine solutions in later iterations to reach lower final fitness values. Its trajectories are generally smoother with smaller fluctuations, demonstrating superior stability and robustness. As the dimensionality increases, many baseline and improved algorithms suffer from slower convergence or premature stagnation, whereas MESPBO maintains strong descending trends and attains higher-precision solutions, with particularly pronounced advantages on complex multimodal and high-dimensional problems. Overall, these results confirm that the proposed multi-strategy enhancements effectively improve population diversity and the exploration–exploitation balance, thereby significantly strengthening global optimization capability and high-dimensional adaptability.

As presented in Table 2, the proposed MESPBO algorithm exhibits outstanding performance on the 10-dimensional CEC2017 benchmark suite. It consistently achieves the best or near-best mean fitness values on the majority of test functions, demonstrating its powerful global optimization capability in low-dimensional search spaces. Moreover, MESPBO reports significantly smaller standard deviations compared with classical algorithms such as PSO, SO, GRO, and SBO, as well as more recent variants including HHWOA, IGWO, MSPBO, QOCSPBO, and SPBO. This evidences the algorithm’s high robustness and stability, ensuring reliable performance across independent runs. Table 3 presents the comparative results of all algorithms tested on the 30-dimensional CEC2017 benchmark suite. As the dimensionality increases, the complexity of the optimization task rises dramatically due to the enlarged search space and more rugged fitness landscapes. Despite these challenges, the proposed MESPBO algorithm maintains excellent optimization performance. MESPBO achieves the best or competitive mean fitness values across a majority of the 30-dimensional test functions, demonstrating that its strong global search capability extends naturally to more complex, higher-dimensional settings. Additionally, the algorithm continues to achieve notably smaller standard deviation values than all other competitors. This indicates that MESPBO remains highly stable and robust even under increased search difficulty. Table 4 reports the experimental results on the 50-dimensional CEC2017 benchmark set. As the dimensionality further increases, the optimization landscape becomes highly rugged and multimodal, greatly intensifying the difficulty of locating global optima. Nevertheless, the proposed MESPBO algorithm continues to deliver remarkable performance. Across the majority of 50-dimensional test functions, MESPBO achieves the lowest mean fitness values, clearly outperforming both traditional and state-of-the-art metaheuristic competitors. Its performance advantage is especially prominent on complex multimodal functions, where maintaining global search ability is crucial. Moreover, MESPBO consistently reports the smallest standard deviations, reaffirming its strong robustness and solution stability even under extremely high-dimensional and complex search scenarios.

Based on the boxplot results of the CEC2017 benchmark functions across 10, 30, and 50 dimensions, the proposed MESPBO algorithm demonstrates consistently superior performance. It exhibits the smallest box heights, short whiskers, and nearly no outliers for most test functions, indicating highly concentrated solution distributions and excellent stability across repeated runs. As the dimensionality increases from 10 to 50, most competing algorithms show noticeable degradation, reflected by significantly expanded box ranges and numerous outliers. In contrast, MESPBO maintains compact distributions and low median fitness values, highlighting its strong capability in handling high-dimensional optimization tasks. For F5, F8, F10, F17, F22, and F29 functions, MESPBO achieves the best or near-best median performance while presenting substantially lower variability than other methods. Overall, these results confirm that MESPBO consistently preserves robust and accurate optimization performance across different dimensional settings and stands out as the most competitive algorithm among all compared methods.

Across the CEC2017 benchmark tests with 10, 30, and 50 dimensions, the experimental results demonstrate that MESPBO consistently delivers superior optimization performance. In terms of mean performance, median values, and distribution stability, MESPBO significantly outperforms the competing algorithms. As the dimensionality increases, most algorithms exhibit larger fluctuations, unstable convergence behaviors, and numerous outliers. In contrast, MESPBO maintains a compact solution distribution, stable convergence, and reliable performance even in high-dimensional scenarios, reflecting its strong adaptability and robustness. Overall, MESPBO demonstrates clear advantages in global search ability, solution stability, and cross-dimensional scalability, making it the most competitive and effective algorithm among all the methods evaluated in this study.

### 4.4. Friedman Mean Rank Test

To further assess the statistical significance of performance differences among algorithms, we employed Friedman’s median rank test for evaluation. Friedman’s test is a nonparametric statistical test designed to detect performance variations across multiple algorithms on various benchmark functions [37]. Unlike parametric tests such as analysis of variance, it does not assume a normal distribution of data, making it particularly suitable for analyzing optimization results where performance values may not follow a Gaussian distribution.

In this test, each algorithm is assigned a ranking for each benchmark function based on its performance, with the best-performing algorithm receiving the lowest average ranking. The ranking of the algorithm across all test functions is then calculated, yielding the average ranking for each algorithm. A lower average ranking indicates better overall performance. Table 5 shows the rankings of each algorithm across various dimensions on the CEC2017 test set. M.R represents the algorithm’s average ranking across 30 test functions, while T.R indicates its final ranking.

As shown in Table 5, the Friedman mean-rank test results demonstrate that MESPBO consistently achieves the best overall performance on the CEC2017 benchmark suite across 10-, 30-, and 50-dimensional settings. Specifically, the mean ranks of MESPBO are 2.00, 1.67, and 1.67 for the three dimensionalities, respectively, which are significantly lower than those of the eleven competing algorithms. Moreover, MESPBO ranks first in total ranking across all dimensions, indicating that it maintains the top position on the majority of test functions. In contrast, traditional algorithms such as PSO and SO, as well as several enhanced variants including IGWO, MSPBO, and QOCSPBO, exhibit relatively high mean ranks and total ranks, reflecting their inferior performance on most test functions. Although algorithms like SPBO and SBOA show comparatively better rankings in some dimensions, their mean ranks remain noticeably higher than those of MESPBO, implying that they cannot compete with MESPBO in terms of overall optimization performance. Overall, the Friedman test results further validate the stability, superiority, and consistency of MESPBO across different dimensional settings. These findings confirm that MESPBO delivers the most competitive and reliable performance among all compared algorithms and is the strongest algorithm in terms of comprehensive optimization capability.

## 5. MESPBO for Feature Selection

In this section, the proposed MESPBO algorithm is applied to the feature selection task to further verify its effectiveness and practicality in real-world optimization scenarios. Feature selection plays a crucial role in machine learning and data mining, as it aims to identify the most informative subset of features that can improve classification accuracy while reducing computational cost and model complexity. However, due to the combinatorial and highly nonlinear nature of the search space, traditional deterministic methods often fail to achieve satisfactory results, especially when dealing with high-dimensional datasets.

To address these challenges, population-based metaheuristic algorithms have been widely adopted for feature selection because of their strong global search capability and flexibility. By leveraging its enhanced exploration–exploitation balance and the reinforcement mechanism introduced through RBMO, the proposed MESPBO algorithm is expected to effectively search for optimal feature subsets and achieve a good trade-off between feature reduction and classification performance. The subsequent experiments evaluate MESPBO against several state-of-the-art metaheuristic algorithms on multiple benchmark datasets to demonstrate its robustness, convergence efficiency, and feature selection quality.

### 5.1. The Proposed MESPBO-KNN

The feature selection (FS) problem refers to the process of selecting an optimal subset of features from an original, high-dimensional feature space to achieve certain optimization objectives [38]. These objectives typically include improving the predictive performance of learning models, enhancing generalization ability, and reducing computational cost and data redundancy. Formally, given an original feature set $F=\left\{f_{1},f_{2},\cdots ,f_{n}\right\}$, the goal of FS is to identify a subset S⊆F that maximizes model accuracy while minimizing the number of selected features.

The K-nearest neighbor (KNN) algorithm is a classic and widely used machine learning classifier that has been successfully applied in many fields [39], including medical image analysis [40], fault diagnosis [41], and natural language processing [42]. KNN performs classification by measuring the similarity between samples using the Euclidean distance. Its mathematical formula is shown in Equation (19).

(19) $$Dis(x_{1}-x_{2})=\sqrt{\sum_{k=1}^{N}{\left(x_{1k}-x_{2k}\right)}^{2}},$$

For feature selection, the ultimate goal is to obtain the highest prediction accuracy by finding the minimum number of features. In this section, we propose a feature selection method called MESPBO-KNN by combining MESPBO with KNN. Assume that the dataset contains D features: $X=\left\{x_{1},x_{2},\cdots ,x_{N}\right\}$, where $x_{i}$ is a D-dimensional feature vector, and y is the response variable. Our goal is to select a subset of features from the original D features to minimize our objective function, which is expressed as Equation (20)

(20) $$\mathrm{Minimize}:\;\alpha \times CER+(1-\alpha )\times \frac{\left|R\right|}{\left|D\right|},$$

where α is a random number sampled from a uniform distribution; CER=1−Accuracy denotes the classification error rate, where Accuracy is calculated by Equation (21); |R| represents the number of selected features; and |D| is the total number of features.

(21) $$Accuracy=\frac{TP+TN}{TP+TN+FP+FN},$$

where TP represents the number of correctly classified positive samples, TN represents the number of correctly classified negative samples, FP indicates the count of false positive instances, and FN refers to positive samples misclassified as negative. The constraints of the optimization problem can be expressed as Equation (22)

(22) $$\mathrm{Subject}\;\mathrm{to}:\;\sum_{j=1}^{D}x_{i,j}\leq K,i\in \{1,\ldots ,N\},$$

where K denotes the maximum number of features allowed to be selected. The decision variables are modeled as Equation (23)

(23) $$\mathrm{With}:\;x_{i,j}\in \{0,1\},i\in \{1,\ldots ,N\},j\in \{1,\ldots ,D\},$$

where $x_{i,j}$ is a binary decision variable indicating whether feature j is selected for sample i: if $x_{i,j}=1$, the feature is selected; otherwise, if $x_{i,j}=0$, the feature is not selected.

### 5.2. Simulation Experiment Analysis

In this section, we use 10 public datasets to evaluate the performance of MESPBO-KNN. It is worth noting that we divide these datasets into three categories: small datasets, medium datasets, and large datasets. Each dataset is divided into training, testing, and validation subsets using cross-validation, and then classified using the KNN classifier. The detailed information of the datasets is shown in Table 6.

**Figure 6:**
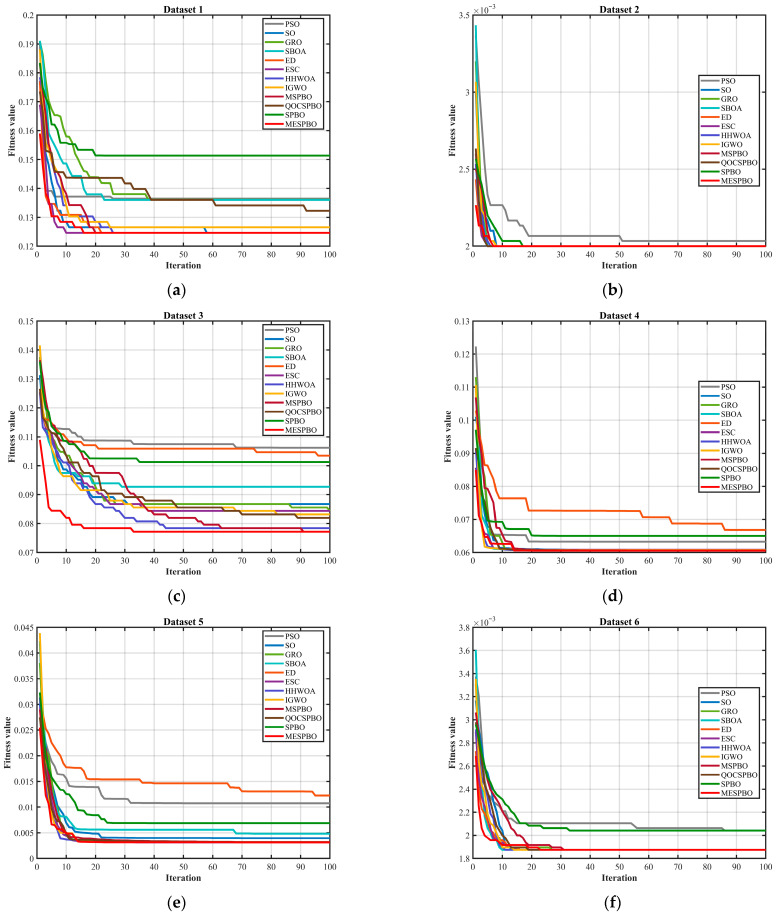

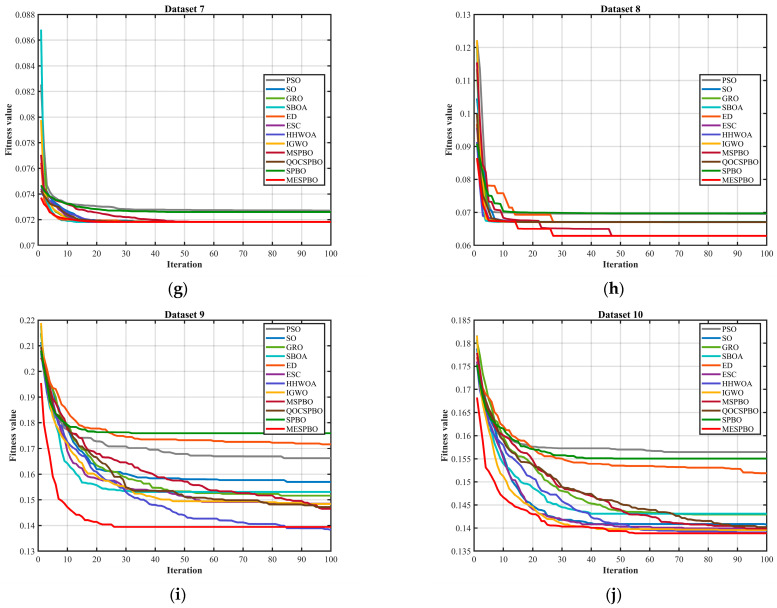
Comparison of convergence speed of different algorithms on Dataset. (**a**) Dataset 1, (**b**) Dataset 2, (**c**) Dataset 3, (**d**) Dataset 4, (**e**) Dataset 5, (**f**) Dataset 6, (**g**) Dataset 7, (**h**) Dataset 8, (**i**) Dataset 9, (**j**) Dataset 10.

In addition, to verify the effectiveness and competitiveness of the proposed MESPBO-KNN algorithm, a series of comparative experiments were conducted against several state-of-the-art algorithms. For fair evaluation and to minimize randomness, the population size and maximum iteration count were fixed at 50 and 100, respectively, and each algorithm was independently run 30 times. The detailed results are presented in Table 7, Table 8 and Table 9. In order to judge the convergence speed of each algorithm, Figure 6 shows the convergence curves of each algorithm on 10 problems.

As illustrated in Figure 6a–j, the convergence curves on ten datasets clearly demonstrate the superiority of MESPBO in both convergence speed and final solution quality. For Datasets 1–6, MESPBO decreases the fitness value much faster than the competing algorithms, typically reaching the lowest or near-lowest level within the first 10–20 iterations, whereas most baselines still exhibit slow descending trends. For more challenging datasets such as Datasets 3, 5, 6, 9, and 10, many algorithms show premature stagnation or weak late-stage improvement, with their curves trapped at relatively high fitness plateaus. In contrast, MESPBO continues to reduce the fitness steadily and finally attains the lowest stable fitness among all methods, indicating stronger global optimization capability. Moreover, for Datasets 7 and 8 where the final performances of different algorithms are close, MESPBO still achieves the best or tied-best terminal fitness with the smallest fluctuation, reflecting excellent stability and consistency. Overall, these convergence results confirm that MESPBO consistently provides faster convergence and better final optimization outcomes across diverse datasets, outperforming PSO, SO, GRO, SBOA, ED, ESC, HHWOA, IGWO, MSPBO, QOCSPBO, and SPBO.

As shown in Table 7, MESPBO achieves superior optimization performance across the ten datasets, consistently obtaining the lowest or near-lowest average fitness values in almost all cases. Specifically, for Datasets 1, 3, 4, 5, 6, 7, 8, 9, and 10, MESPBO attains the best or tied-best mean performance among all algorithms, indicating its strong ability to adapt to various data characteristics and deliver high-quality solutions. In addition, MESPBO exhibits the smallest standard deviations across all datasets, with values significantly lower than those of other competing algorithms. This demonstrates excellent consistency over multiple runs and highlights the algorithm’s strong stability. In contrast, several enhanced algorithms such as HHWOA, MSPBO, and QOCSPBO occasionally produce comparable mean fitness on certain datasets but generally suffer from larger standard deviations, implying unstable convergence. Traditional methods like PSO, SO, and GRO show relatively higher average fitness values on many datasets, reflecting their tendency to be trapped in local optima and their limited overall performance. Overall, the statistical results in Table 7 further confirm the robustness, reliability, and comprehensive superiority of MESPBO. The algorithm not only delivers the best solution quality but also maintains remarkable stability across diverse datasets, making it the most balanced and effective optimization method among all competitors.

According to the accuracy comparison in Table 8, MESPBO achieves overall leading performance across the ten datasets. For Dataset 2, Dataset 4, Dataset 6, and Dataset 7, all algorithms obtain almost identical accuracies (100% for Datasets 4/6/7 and 97.1% for Dataset 2), indicating that these datasets are relatively easy and MESPBO maintains equally optimal performance. On more discriminative and challenging datasets, MESPBO shows clearer superiority: it attains the highest accuracies of 95.81%, 93.33%, 81.5%, and 81.5% on Datasets 5, 8, 9, and 10, respectively, outperforming all competitors; it also achieves the best or near-best result on Dataset 3 with 89.0%. Although MESPBO is slightly below the top accuracy on Dataset 1 (with a marginal gap of about 0.2%), its performance remains within the top tier. Overall, MESPBO delivers the best accuracies on most complex datasets while preserving optimal consistency on easier ones, demonstrating strong generalization ability and stable classification performance.

As reported in Table 9, MESPBO generally selects fewer or an equal number of features while maintaining competitive classification performance, demonstrating superior feature reduction capability. For Datasets 1–4, the average number of selected features is very close across all algorithms, and MESPBO achieves the same minimal level as the best competitors, indicating that it does not introduce redundant features on relatively simple datasets. On more challenging datasets (Datasets 5–8), MESPBO shows a clearer advantage by selecting notably fewer features; for instance, it chooses about 2.1 features on Dataset 5, which is lower than PSO, SO, GRO, and ESC (typically ranging from about 2.2 to 3.6). For high-dimensional feature datasets such as Dataset 9 and Dataset 10, MESPBO again yields the smallest or tied-smallest feature subset (around 5.7/6 features), significantly fewer than several competitors. Overall, these results confirm that MESPBO can obtain compact and effective feature subsets across diverse datasets, reducing model complexity while preserving search effectiveness, and thus provides a strong and stable feature selection performance.

Overall, the convergence curves and Table 7, Table 8 and Table 9 consistently show that MESPBO delivers the best comprehensive performance across the ten datasets. It achieves the lowest mean fitness values with the smallest standard deviations on most datasets, indicating superior solution quality and robustness. In terms of classification accuracy, MESPBO attains the best or near-best results on challenging datasets while matching the optimal performance on easier ones. Moreover, it generally selects the smallest or tied-smallest number of features, effectively reducing redundancy and model complexity without sacrificing accuracy. In summary, MESPBO demonstrates clear advantages in convergence efficiency, optimization reliability, accuracy, and feature reduction capability, confirming its effectiveness for feature selection and classification optimization tasks.

### 5.3. MESPBO for Photovoltaic Model Parameter Extraction

#### 5.3.1. Single Diode Model (SDM)

**Figure 7:**
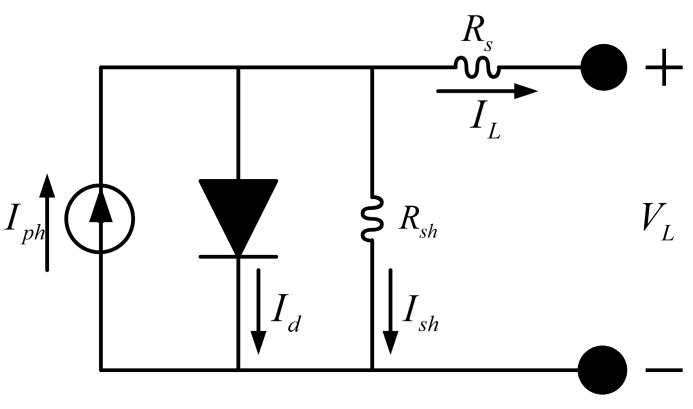
Equivalent circuit of the single diode model.

The Single Diode Model (SDM) is one of the most classic and commonly used equivalent circuit models for photovoltaic (PV) devices and arrays. It accurately characterizes the nonlinear I-V/P-V characteristics of the cell with fewer parameters, achieving a good balance between accuracy and complexity. Therefore, it is widely used in engineering simulation, performance evaluation, and control design. Furthermore, SDM parameters have clear physical correspondences, reflecting the impact of factors such as temperature, irradiance aging, and shading on the internal mechanisms and external output of the device. This makes it an important tool for understanding PV degradation mechanisms, diagnosing faults, and conducting reliability analysis. Accurate SDM parameter identification is fundamental to many critical applications, such as maximum power point tracking (MPPT) algorithm design, inverter and grid-connected control, and PV system energy prediction and scheduling optimization. Inaccurate models or parameters will directly lead to power estimation errors, decreased control efficiency, and even system instability. In practical applications, PV systems often operate under dynamic environments (rapid temperature/irradiance changes, partial shading, and multi-peak characteristics). Researching high-precision and robust parameter extraction methods for single-diode (SDM) models helps improve the model’s generalization ability and real-time availability under complex conditions. Therefore, in this section, we investigate parameter extraction for single-diode models. The equivalent circuit of the single-diode model is shown in Figure 7.

The single-diode model comprises a current source that represents the photo-generated current induced by solar irradiation, a diode that characterizes the PN junction behavior of the semiconductor, a series resistance $R_{s}$ reflecting the ohmic losses of electrodes, interconnections, and materials, and a shunt resistance $R_{sh}$ accounting for leakage paths through the semiconductor structure. The output current can be represented by Equation (24).

(24) $$I_{out}=I_{ph}-I_{d}-I_{sh},$$

where $I_{ph}$, $I_{d}$, and $I_{sh}$ denote the photocurrent, the diode current, and the current through the shunt resistor, respectively. The diode and shunt currents can be expressed as follows:

(25) $$I_{d}=I_{o}\cdot \left\{\exp\left[\frac{q\cdot \left(V_{\mathrm{out}}+R_{s}\cdot I_{\mathrm{out}}\right)}{a\cdot k\cdot T}\right]-1\right\},$$

(26) $$I_{\mathrm{sh}}=\frac{V_{\mathrm{out}}+R_{s}\cdot I_{\mathrm{out}}}{R_{sh}},$$

where $I_{o}$ is the diode reverse saturation current, a is the diode ideality factor, k is the Boltzmann constant $\left(1.3806503\times 10^{-23}\;J\cdot K^{-1}\right)$, q is the electron charge $\left(1.60217646\times 10^{-19}\;C\right)$, and T ambient temperature in Kelvin.

Substituting Equations (25) and (26) into Equation (24) yields the output current–voltage relationship of the single-diode model:

(27) $$I_{out}=I_{ph}-I_{o}\cdot \left\{\exp\left[\frac{q\cdot \left(V_{\mathrm{out}}+R_{s}\cdot I_{\mathrm{out}}\right)}{a\cdot k\cdot T}\right]-1\right\}-\frac{V_{\mathrm{out}}+R_{s}\cdot I_{\mathrm{out}}}{R_{sh}},$$

Thus, the single-diode model is fully characterized by five parameters: $\left(I_{ph},I_{o},a,R_{s},R_{sh}\right)$.

The unknown parameters are obtained by casting their estimation as an optimization task, in which an objective function g quantifies the mismatch between the measured experimental values and the model’s predicted outputs. The optimization procedure seeks to minimize this mismatch over a predefined search space, thereby yielding the best-fitting set of parameters. Commonly adopted error formulations for this purpose are listed below:

(28) $$\left\{\begin{array}{c} g\left(V_{\mathrm{out}},I_{\mathrm{out}},y\right)=N_{p}\cdot I_{ph}-N_{p}\cdot I_{o}\cdot \left\{exp\left[\frac{q\cdot \left(\frac{V_{\mathrm{out}}}{N_{s}}+R_{s}\cdot \frac{I_{\mathrm{out}}}{N_{p}}\right)}{a\cdot k\cdot T}\right]-1\right\}-\\ N_{p}\cdot \frac{\frac{V_{\mathrm{out}}}{N_{s}}+R_{s}\cdot \frac{I_{\mathrm{out}}}{N_{p}}}{R_{\mathrm{sh}}}-I_{\mathrm{out}}\\ y=\left(I_{ph},I_{o},a,R_{s},R_{\mathrm{sh}}\right) \end{array}\right.,$$

where set $N_{s}=1$ and $N_{p}=1$. The total discrepancy between the experimental I−V curve and the model prediction is assessed using the root mean square error (RMSE):

(29) $$RMSE\left(y\right)=\sqrt{\frac{\sum_{n=1}^{N}{\left(V_{\mathrm{out},n},I_{\mathrm{out},n},y\right)}^{2}}{N}},$$

where N is the total number of measured data points $\left(V_{\mathrm{out},n},I_{\mathrm{out},n}\right)$.

#### 5.3.2. Experimental Parameter Setting and Simulation Analysis

In this section, the effectiveness of the proposed MESPBO algorithm in photovoltaic model parameter identification is thoroughly evaluated. We first provide a concise description of the experimental setup and related parameters. Subsequently, MESPBO is employed to estimate the unknown variables of the single-diode model (SDM). The detailed settings and procedures are presented as follows:

(1) Experimental Parameter setting

Experimental measurements were obtained from a Photowatt-PWP 201 photovoltaic module comprising 36 polycrystalline silicon cells connected in series. At an operating temperature of 33 °C and a solar irradiance of 1000 W/m^2^, a total of 26 current–voltage (I–V) data points were recorded. These data were used to identify the unknown parameters of both the SDM and DDM for RTC France PV cells, and the resulting estimates were subsequently benchmarked against those produced by other state-of-the-art optimization methods.

All compared algorithms were coded in MATLAB 2024b and run on a personal computer equipped with a 2.5 GHz CPU, 16 GB RAM, and Windows 11. For each problem, every algorithm was executed independently 30 times, using a population size of 50 and a maximum of 1000 iterations. To emphasize performance differences and verify their statistical reliability, the Wilcoxon rank-sum test was adopted. The feasible ranges of the unknown parameters for each model are listed in Table 10, where Lb and Ub denote the lower and upper bounds, respectively.

As noted above, the root mean square error (RMSE) provides a simple and effective measure of the discrepancy between experimental observations and model simulations. A smaller RMSE indicates a tighter match between the calculated and measured data, thereby demonstrating the algorithm’s stronger ability to identify the unknown parameters of the photovoltaic system. In other words, the extracted diode model can more faithfully capture the real operating characteristics of solar cells and PV modules. Therefore, reducing this error is of vital importance.

Moreover, the absolute error (IAE) and relative error (RE) are adopted to evaluate the deviation at each measured voltage point, which are defined as follows:

(30) $$IAE=\left|I_{measure}-I_{simulate}\right|,$$

(31) $$RE=\frac{I_{measure}-I_{simulate}}{I_{measure}},$$

(2) Experimental Analysis of SDM

**Figure 8:**
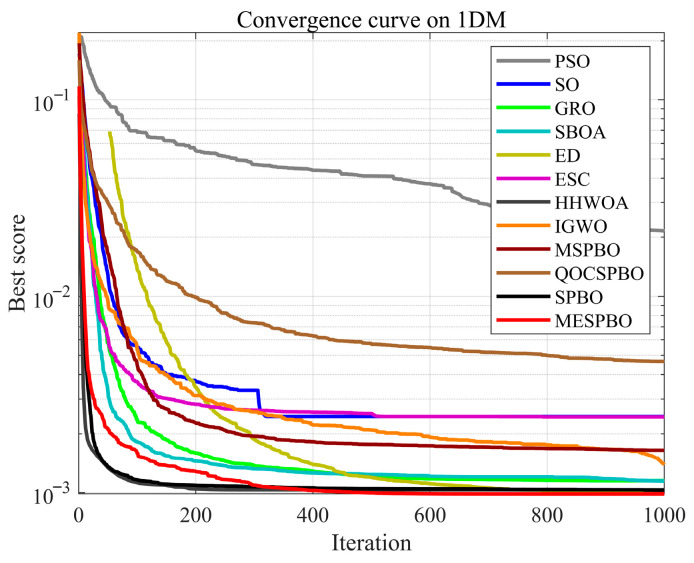
The convergence curve of MESPBO and other algorithms on the SDM.

In this subsection, we conducted experimental analysis using MESPBO and 11 other comparison algorithms. The experimental results are shown in Table 11, Meanwhile, Figure 8 shows its convergence curve. From the convergence curves on the 1DM model, all algorithms reduce the objective rapidly at the beginning, but their convergence rates and final precisions differ markedly. MESPBO achieves the steepest early decrease, driving the best score down to about the 10^−3^ level within the first tens of iterations, and stabilizes after roughly 200 iterations with the lowest final error among all competitors. This indicates both fast convergence and high solution accuracy. Algorithms such as SPBO, HHWOA, GRO, SBOA, and ED also keep improving toward the 10^−3^ range, but they converge more slowly or exhibit mid/late-stage plateaus, reflecting weaker exploitation or stability than MESPBO. In contrast, IGWO, MSPBO, ESC, and SO stagnate at higher error levels, suggesting premature convergence. PSO and QOCSPBO show the slowest convergence and the highest final errors, implying insufficient global exploration and fine local search for this parameter-identification task. Overall, MESPBO combines the fastest early descent with the best final precision and minimal stagnation, confirming that its multi-strategy enhancements significantly improve convergence speed, accuracy, and robustness on the 1DM PV model.

**Figure 9:**
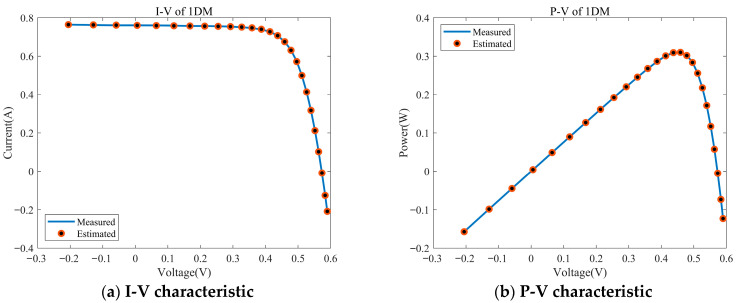
The I-V and P-V characteristics of the estimated SDM identified by MESPBO. (**a**) I-V of 1DM, (**b**) P-V of 1DM.

Figure 9 shows the I-V and P-V fitting results of MESPBO after parameter identification of the single diode model (1DM): the estimated curve (red circle) almost coincides with the measured curve (blue line) over the entire voltage range. The current plateau, inflection point/knee point position in the short-circuit region and the rapid drop segment near the open-circuit voltage are all aligned, indicating that the photocurrent, series and parallel resistance, diode parameters, etc., are accurately identified. The voltage position and peak height of the maximum power point in the corresponding P-V curve are also consistent with the measured values. The power drop trend after the peak is well matched, indicating that the algorithm can not only accurately fit the current–voltage characteristics, but also reliably predict the power output and MPP parameters, verifying the high accuracy and stability of MESPBO in 1DM parameter identification.

Table 12 reports the point-wise absolute errors of current and power obtained by MESPBO under the SDM. The overall pattern indicates highly accurate and stable fitting across the whole voltage range: the current errors (IAE_I) are mostly within the 10^−4^~10^−3^ order, showing only a slight increase in the knee/high-voltage sensitive region, while still remaining very small; the power errors (IAE_P) are even smaller, generally in the 10^−5^~10^−4^ range, with only minor fluctuations around the peak-power area and no systematic deviation. These results confirm that the SDM parameters identified by MESPBO can reconstruct the I–V and P–V characteristics with excellent global accuracy and robustness, achieving the smallest errors in the low-voltage region and maintaining low errors even near the knee point and MPP.

## 6. Summary and Limitations

In this paper, a novel metaheuristic algorithm called multi-strategy-enhanced student psychology-based optimization (MESPBO) was proposed to improve the optimization capability of the SPBO. By integrating three strategies—hybrid heuristic population initialization, adaptive dual-learning position update, and hybrid opposition-based reflective boundary control—MESPBO effectively enhances population diversity, improves convergence accuracy, and prevents premature stagnation. Extensive experiments conducted on the CEC2017 benchmark suite under 10-, 30-, and 50-dimensional scenarios demonstrated that MESPBO consistently outperforms eleven state-of-the-art optimization algorithms in terms of convergence speed, solution precision, and robustness. Furthermore, its successful application to feature selection tasks confirmed that MESPBO can efficiently reduce redundant features while maintaining or improving classification accuracy, thus proving its strong generalization ability and applicability in both continuous and combinatorial optimization domains.

Although MESPBO demonstrates strong performance and generalization ability across both benchmark optimization and feature selection tasks, several inherent limitations should be acknowledged. First, due to the integration of multiple enhancement strategies, the overall algorithmic structure becomes more complex than that of the original SPBO. This increased computational overhead may lead to relatively higher time consumption when handling extremely large populations or very high-dimensional optimization problems. Second, similar to most population-based metaheuristics, MESPBO still relies on stochastic search operators, which may introduce performance fluctuations across independent runs. Although robustness has been significantly improved, absolute consistency cannot be guaranteed. Third, the algorithm includes several hyperparameters related to learning mechanisms and boundary control, and while their default settings work well across various problems, their sensitivity on domain-specific tasks may still require careful tuning.

## Figures and Tables

**Table 1:** Compare algorithm parameter settings.

Algorithms	Name of the Parameter	Value of the Parameter
PSO	Vmax, wMax, wMin, c1, c2	6, 0.9, 0.6, 2, 2
SO	c1, c2, c3	0.5, 0.05, 2
GRO	sigma_initial	2
SBOA	tt	0.5
ED	ishow	250
ESC	eliteSize, beta_base	5, 1.5
HHWOA	w	3
IGWO	a	2
MSPBO	U, R1, R2	0.5, 0.33, 0.66
QOCSPBO	jumpRate	0.2

**Table 2:** Experimental results of CEC2017 (dim = 10).

ID	Metric	PSO	SO	GRO	SBOA	ED	ESC	HHWOA	IGWO	MSPBO	QOCSPBO	SPBO	MESPBO
F1	mean	2.9814 × 10^3^	3.3019 × 10^3^	1.8098 × 10^3^	2.2149 × 10^3^	2.4692 × 10^3^	1.3422 × 10^3^	3.7052 × 10^2^	1.7027 × 10^4^	1.4412 × 10^3^	1.1691 × 10^8^	**1.5116 × 10^2^**	1.8885 × 10^2^
	std	4.0299 × 10^3^	7.8603 × 10^3^	1.9065 × 10^3^	2.3589 × 10^3^	1.4839 × 10^3^	1.1709 × 10^3^	1.4817 × 10^3^	5.5602 × 10^3^	1.8378 × 10^3^	2.6019 × 10^8^	8.0474 × 10^1^	8.1775 × 10^1^
F2	mean	**2.0000 × 10^2^**	2.0760 × 10^2^	6.1530 × 10^2^	**2.0000 × 10^2^**	**2.0000 × 10^2^**	**2.0000 × 10^2^**	2.0043 × 10^2^	**2.0000 × 10^2^**	2.5830 × 10^4^	1.0809 × 10^7^	2.0000 × 10^2^	2.0000 × 10^2^
	std	**0.0000 × 10^0^**	3.2625 × 10^1^	6.7882 × 10^2^	**0.0000 × 10^0^**	**0.0000 × 10^0^**	**0.0000 × 10^0^**	6.2606 × 10^−1^	**0.0000 × 10^0^**	6.8349 × 10^4^	2.5789 × 10^7^	**0.0000 × 10^0^**	**0.0000 × 10^0^**
F3	mean	3.0000 × 10^2^	3.0000 × 10^2^	3.0034 × 10^2^	3.0000 × 10^2^	3.3974 × 10^2^	3.0102 × 10^2^	**3.0000 × 10^2^**	3.0005 × 10^2^	1.5391 × 10^3^	2.1619 × 10^3^	2.9897 × 10^3^	3.0000 × 10^2^
	std	2.9513 × 10^−4^	2.9154 × 10^−3^	1.2123 × 10	7.2192 × 10^−12^	5.3821 × 10^1^	2.8371 × 10	**5.1711 × 10^−14^**	3.6635 × 10^−2^	5.7748 × 10^2^	8.8096 × 10^2^	1.8069 × 10^3^	5.0623 × 10^−14^
F4	mean	4.0579 × 10^2^	4.0377 × 10^2^	4.0435 × 10^2^	**4.0207 × 10^2^**	4.0216 × 10^2^	4.0453 × 10^2^	4.0221 × 10^2^	4.0217 × 10^2^	4.0661 × 10^2^	4.5913 × 10^2^	4.0025 × 10^2^	4.0046 × 10^2^
	std	1.1816 × 10^1^	1.9050 × 10^0^	1.9667 × 10^0^	9.0036 × 10^−1^	5.0678 × 10^−1^	**2.6380 × 10^−1^**	1.1082 × 10^1^	9.0710 × 10^−1^	6.2636 × 10^−1^	5.7352 × 10^1^	3.5411 × 10^−1^	2.4265 × 10^−1^
F5	mean	5.3582 × 10^2^	5.1181 × 10^2^	5.0769 × 10^2^	5.0809 × 10^2^	5.1328 × 10^2^	**5.0426 × 10^2^**	5.1682 × 10^2^	5.0678 × 10^2^	5.1721 × 10^2^	5.5706 × 10^2^	5.0517 × 10^2^	5.0425 × 10^2^
	std	1.1597 × 10^1^	4.3230 × 10^0^	4.0976 × 10^0^	3.5127 × 10^0^	3.7001 × 10^0^	**2.1595 × 10** ^0^	7.6273 × 10^0^	5.3774 × 10^0^	6.8634 × 10^0^	1.9745 × 10^1^	1.3427 × 10^0^	8.9587 × 10^−1^
F6	mean	6.0716 × 10^2^	6.0006 × 10^2^	6.0000 × 10^2^	**6.0000 × 10^2^**	6.0000 × 10^2^	6.0000 × 10^2^	6.0033 × 10^2^	6.0005 × 10^2^	6.0000 × 10^2^	6.3391 × 10^2^	6.0000 × 10^2^	6.0000 × 10^2^
	std	6.2732 × 10^0^	1.9504 × 10^−1^	3.3782 × 10^−3^	5.5268 × 10^−6^	1.5177 × 10^−4^	**4.6974 × 10^−6^**	9.7174 × 10^−1^	1.2289 × 10^−2^	1.7287 × 10^−4^	1.0239 × 10^1^	1.0342 × 10^−13^	7.6117 × 10^−14^
F7	mean	7.2317 × 10^2^	7.2568 × 10^2^	7.1808 × 10^2^	7.1789 × 10^2^	7.2233 × 10^2^	**7.1462 × 10^2^**	7.2262 × 10^2^	7.2167 × 10^2^	7.3359 × 10^2^	7.6940 × 10^2^	7.1506 × 10^2^	7.1081 × 10^2^
	std	4.8619 × 10^0^	7.3540 × 10^0^	2.6763 × 10^0^	5.7604 × 10^0^	3.0334 × 10^0^	**1.6277 × 10^0^**	5.6667 × 10^0^	8.2655 × 10^0^	7.6246 × 10^0^	1.7869 × 10^1^	1.3952 × 10^0^	3.2041 × 10^0^
F8	mean	8.2043 × 10^2^	8.1316 × 10^2^	8.0899 × 10^2^	8.0909 × 10^2^	8.1322 × 10^2^	**8.0368 × 10^2^**	8.1496 × 10^2^	8.0740 × 10^2^	8.1619 × 10^2^	8.2864 × 10^2^	8.0551 × 10^2^	8.0331 × 10^2^
	std	7.2160 × 10^0^	6.0902 × 10^0^	3.2053 × 10^0^	4.3665 × 10^0^	3.2359 × 10^0^	**1.5683 × 10^0^**	7.1818 × 10^0^	5.4027 × 10^0^	6.2505 × 10^0^	6.9581 × 10^0^	1.8793 × 10^0^	7.8205 × 10^−1^
F9	mean	9.0000 × 10^2^	9.0022 × 10^2^	9.0000 × 10^2^	**9.0000 × 10^2^**	9.0003 × 10^2^	9.0000 × 10^2^	9.0028 × 10^2^	9.0000 × 10^2^	9.0000 × 10^2^	1.3009 × 10^3^	9.0000 × 10^2^	9.0000 × 10^2^
	std	7.0853 × 10^−7^	5.7142 × 10^−1^	2.5516 × 10^−7^	**9.4412 × 10^−14^**	9.3033 × 10^−2^	8.2697 × 10^−9^	4.1145 × 10^−1^	4.1183 × 10^−4^	2.2169 × 10^−3^	1.7234 × 10^2^	3.6464 × 10^−6^	**0.0000 × 10^0^**
F10	mean	1.8351 × 10^3^	1.4772 × 10^3^	1.3651 × 10^3^	1.3071 × 10^3^	1.7283 × 10^3^	**1.1977 × 10^3^**	1.7373 × 10^3^	1.3397 × 10^3^	1.8416 × 10^3^	2.0960 × 10^3^	1.1607 × 10^3^	1.1192 × 10^3^
	std	3.0903 × 10^2^	1.8638 × 10^2^	2.0713 × 10^2^	1.7428 × 10^2^	1.9398 × 10^2^	**1.0851 × 10^2^**	3.4245 × 10^2^	3.7366 × 10^2^	2.2667 × 10^2^	2.2106 × 10^2^	9.0718 × 10^1^	4.3279 × 10^1^
F11	mean	1.1370 × 10^3^	1.1093 × 10^3^	1.1037 × 10^3^	1.1036 × 10^3^	1.1041 × 10^3^	**1.1026 × 10^3^**	1.1168 × 10^3^	1.1044 × 10^3^	1.1067 × 10^3^	1.2519 × 10^3^	1.1022 × 10^3^	1.1005 × 10^3^
	std	1.9167 × 10^1^	5.0477 × 10^0^	**1.2459 × 10^0^**	1.4220 × 10^0^	2.3872 × 10^0^	1.4531 × 10^0^	1.6768 × 10^1^	2.1990 × 10^0^	2.7680 × 10^0^	9.5849 × 10^1^	1.1243 × 10^0^	4.1158 × 10^−1^
F12	mean	1.7014 × 10^4^	1.3361 × 10^4^	1.5602 × 10^4^	1.6323 × 10^4^	5.1249 × 10^4^	1.1953 × 10^4^	**1.3330 × 10^3^**	1.8918 × 10^4^	1.4100 × 10^5^	4.1247 × 10^6^	2.1198 × 10^4^	3.5939 × 10^3^
	std	1.0552 × 10^4^	1.0458 × 10^4^	1.2176 × 10^4^	1.6828 × 10^4^	2.9166 × 10^4^	9.0781 × 10^3^	**1.5341 × 10^2^**	1.6807 × 10^4^	1.5868 × 10^5^	3.5646 × 10^6^	1.6353 × 10^4^	8.8142 × 10^2^
F13	mean	6.3821 × 10^3^	4.7231 × 10^3^	2.1871 × 10^3^	2.2813 × 10^3^	3.6262 × 10^3^	6.0238 × 10^3^	**1.3045 × 10^3^**	2.1359 × 10^3^	4.4067 × 10^3^	1.2331 × 10^4^	1.5060 × 10^3^	1.3769 × 10^3^
	std	5.9593 × 10^3^	3.3906 × 10^3^	7.7793 × 10^2^	1.3675 × 10^3^	1.5563 × 10^3^	6.5014 × 10^3^	**2.6734 × 10^0^**	5.4946 × 10^2^	2.7957 × 10^3^	7.5568 × 10^3^	2.3749 × 10^2^	3.5321 × 10^1^
F14	mean	1.6978 × 10^3^	1.4891 × 10^3^	1.4383 × 10^3^	1.4315 × 10^3^	1.5168 × 10^3^	2.1039 × 10^3^	**1.4238 × 10^3^**	1.4512 × 10^3^	1.6156 × 10^3^	2.4446 × 10^3^	1.4800 × 10^3^	1.4179 × 10^3^
	std	4.5901 × 10^2^	4.8873 × 10^1^	**1.0416 × 10^1^**	1.0730 × 10^1^	7.0773 × 10^1^	2.2352 × 10^3^	1.9862 × 10^1^	1.1742 × 10^1^	1.6062 × 10^2^	9.3036 × 10^2^	6.1648 × 10^1^	6.3990 × 10
F15	mean	2.1077 × 10^3^	1.6639 × 10^3^	1.5611 × 10^3^	1.5223 × 10^3^	1.5579 × 10^3^	1.8968 × 10^3^	**1.5170 × 10^3^**	1.5274 × 10^3^	2.1181 × 10^3^	7.8864 × 10^3^	1.5971 × 10^3^	1.5087 × 10^3^
	std	7.7348 × 10^2^	1.1109 × 10^2^	5.2152 × 10^1^	2.1792 × 10^1^	4.1510 × 10^1^	7.7996 × 10^2^	4.4404 × 10^1^	**1.3013 × 10^1^**	4.4678 × 10^2^	2.7619 × 10^3^	1.1985 × 10^2^	3.1644 × 10^0^
F16	mean	1.8504 × 10^3^	1.6932 × 10^3^	1.6325 × 10^3^	1.6116 × 10^3^	1.6054 × 10^3^	1.6168 × 10^3^	1.6450 × 10^3^	**1.6038 × 10^3^**	1.6250 × 10^3^	1.9686 × 10^3^	1.6074 × 10^3^	1.6013 × 10^3^
	std	1.0783 × 10^2^	9.6429 × 10^1^	5.4797 × 10^1^	4.5520 × 10^1^	1.2992 × 10^1^	3.3072 × 10^1^	5.3681 × 10^1^	**1.6456 × 10**	1.5599 × 10^1^	1.0766 × 10^2^	2.3212 × 10^1^	2.8320 × 10^−1^
F17	mean	1.7649 × 10^3^	1.7356 × 10^3^	1.7278 × 10^3^	1.7178 × 10^3^	1.7069 × 10^3^	**1.7047 × 10^3^**	1.7224 × 10^3^	1.7322 × 10^3^	1.7437 × 10^3^	1.7800 × 10^3^	1.7008 × 10^3^	1.7173 × 10^3^
	std	3.4152 × 10^1^	2.4262 × 10^1^	1.0797 × 10^1^	1.0540 × 10^1^	**2.2991 × 10^0^**	7.0844 × 10^0^	1.3511 × 10^1^	9.8757 × 10^0^	1.0337 × 10^1^	2.1877 × 10^1^	7.1941 × 10^−1^	6.2297 × 10^0^
F18	mean	1.1272 × 10^4^	5.9195 × 10^3^	2.8207 × 10^3^	5.3461 × 10^3^	5.4902 × 10^3^	7.4367 × 10^3^	**1.8068 × 10^3^**	4.9668 × 10^3^	7.1777 × 10^3^	1.6427 × 10^4^	2.5954 × 10^3^	2.1796 × 10^3^
	std	7.2253 × 10^3^	4.6359 × 10^3^	9.4287 × 10^2^	2.5627 × 10^3^	1.4442 × 10^3^	7.4005 × 10^3^	**9.1106 × 10^1^**	3.4350 × 10^3^	2.8988 × 10^3^	1.2650 × 10^4^	7.2833 × 10^2^	1.3448 × 10^2^
F19	mean	3.0216 × 10^3^	2.1863 × 10^3^	1.9692 × 10^3^	1.9171 × 10^3^	1.9275 × 10^3^	4.7177 × 10^3^	**1.9001 × 10^3^**	1.9230 × 10^3^	2.6122 × 10^3^	1.4083 × 10^4^	1.9880 × 10^3^	1.9067 × 10^3^
	std	1.8474 × 10^3^	5.4463 × 10^2^	1.0842 × 10^2^	1.2157 × 10^1^	2.4497 × 10^1^	4.4077 × 10^3^	**2.7798 × 10^−1^**	1.1630 × 10^1^	7.8364 × 10^2^	9.5644 × 10^3^	1.6802 × 10^2^	1.7189 × 10^0^
F20	mean	2.0877 × 10^3^	2.0256 × 10^3^	2.0163 × 10^3^	2.0061 × 10^3^	2.0058 × 10^3^	**2.0002 × 10^3^**	2.0093 × 10^3^	2.0248 × 10^3^	2.0187 × 10^3^	2.1800 × 10^3^	2.0002 × 10^3^	2.0010 × 10^3^
	std	5.4271 × 10^1^	2.7431 × 10^1^	1.9328 × 10^1^	8.4835 × 10	4.3121 × 10	**4.7606 × 10^−1^**	1.0720 × 10^1^	6.6342 × 10	1.2386 × 10^1^	5.5017 × 10^1^	2.6765 × 10^−1^	7.0716 × 10^−1^
F21	mean	2.3207 × 10^3^	2.3090 × 10^3^	2.2465 × 10^3^	2.2914 × 10^3^	**2.2158 × 10^3^**	2.2978 × 10^3^	2.2809 × 10^3^	2.2776 × 10^3^	2.2726 × 10^3^	2.2375 × 10^3^	2.2037 × 10^3^	2.2000 × 10^3^
	std	4.2719 × 10^1^	2.0798 × 10^1^	5.3799 × 10^1^	4.1424 × 10^1^	3.9269 × 10^1^	2.8473 × 10^1^	5.3017 × 10^1^	4.8106 × 10^1^	3.3858 × 10^1^	**1.8187 × 10^1^**	1.9453 × 10^1^	1.2942 × 10^−6^
F22	mean	2.3010 × 10^3^	2.3017 × 10^3^	2.2970 × 10^3^	2.2978 × 10^3^	**2.2879 × 10^3^**	2.3004 × 10^3^	2.2994 × 10^3^	2.2981 × 10^3^	2.3018 × 10^3^	2.3351 × 10^3^	2.2582 × 10^3^	2.2446 × 10^3^
	std	1.3015 × 10^1^	5.9380 × 10^−1^	1.8325 × 10^1^	1.4534 × 10^1^	3.1827 × 10^1^	**2.7929 × 10^−1^**	1.2660 × 10^1^	2.6677 × 10^1^	7.7753 × 10^0^	1.4254 × 10^1^	3.5382 × 10^1^	4.3063 × 10^1^
F23	mean	2.6864 × 10^3^	2.6149 × 10^3^	2.6080 × 10^3^	2.6103 × 10^3^	2.6141 × 10^3^	**2.6061 × 10^3^**	2.6190 × 10^3^	2.6108 × 10^3^	2.6149 × 10^3^	2.6646 × 10^3^	2.6039 × 10^3^	2.6065 × 10^3^
	std	2.6026 × 10^1^	5.6227 × 10^0^	3.0176 × 10^0^	4.0292 × 10^0^	2.8214 × 10^0^	**2.3841 × 10^0^**	9.4645 × 10^0^	7.4644 × 10^0^	6.6855 × 10^0^	2.7099 × 10^1^	3.5565 × 10^1^	9.3062 × 10^−1^
F24	mean	2.7832 × 10^3^	2.7430 × 10^3^	2.6710 × 10^3^	2.6905 × 10^3^	**2.5815 × 10^3^**	2.7363 × 10^3^	2.7449 × 10^3^	2.7265 × 10^3^	2.7422 × 10^3^	2.6877 × 10^3^	2.5199 × 10^3^	2.6166 × 10^3^
	std	9.1167 × 10^1^	4.8112 × 10^0^	1.0492 × 10^2^	9.6898 × 10^1^	1.0098 × 10^2^	**2.9374 × 10^0^**	6.9177 × 10^0^	4.3174 × 10^1^	2.6718 × 10^1^	1.2556 × 10^2^	6.3539 × 10^1^	1.1864 × 10^2^
F25	mean	2.9242 × 10^3^	2.9258 × 10^3^	2.9050 × 10^3^	2.9167 × 10^3^	**2.8583 × 10^3^**	2.9320 × 10^3^	2.9293 × 10^3^	2.8996 × 10^3^	2.9390 × 10^3^	2.9564 × 10^3^	2.7554 × 10^3^	2.8979 × 10^3^
	std	2.2794 × 10^1^	2.2640 × 10^1^	1.5558 × 10^1^	2.2695 × 10^1^	7.1246 × 10^1^	2.2564 × 10^1^	2.3294 × 10^1^	**8.3610 × 10^0^**	1.3779 × 10^1^	1.8307 × 10^1^	1.4080 × 10^2^	1.3630 × 10^−1^
F26	mean	3.0505 × 10^3^	3.0843 × 10^3^	2.8633 × 10^3^	2.9020 × 10^3^	**2.8460 × 10^3^**	2.9386 × 10^3^	2.9904 × 10^3^	2.9000 × 10^3^	2.9685 × 10^3^	3.1960 × 10^3^	2.6743 × 10^3^	2.8509 × 10^3^
	std	2.5484 × 10^2^	1.9785 × 10^2^	9.2786 × 10^1^	4.1088 × 10^1^	6.8196 × 10^1^	1.6053 × 10^2^	1.8862 × 10^2^	**2.5704 × 10^−3^**	3.3328 × 10^1^	3.4205 × 10^2^	9.8219 × 10^1^	8.1519 × 10^1^
F27	mean	3.1357 × 10^3^	3.1021 × 10^3^	3.0926 × 10^3^	3.0904 × 10^3^	3.0932 × 10^3^	3.0901 × 10^3^	3.0947 × 10^3^	**3.0894 × 10^3^**	3.0905 × 10^3^	3.1459 × 10^3^	3.0928 × 10^3^	3.0889 × 10^3^
	std	5.6496 × 10^1^	4.9836 × 10^0^	2.5674 × 10^0^	1.4881 × 10^0^	2.4482 × 10^0^	1.0209 × 10^0^	3.8125 × 10^0^	**3.5394 × 10^−1^**	1.2186 × 10^0^	4.7537 × 10^1^	2.4456 × 10^0^	3.6842 × 10^−1^
F28	mean	3.1593 × 10^3^	3.3659 × 10^3^	3.1313 × 10^3^	3.1783 × 10^3^	**3.0775 × 10^3^**	3.3036 × 10^3^	3.2670 × 10^3^	3.2103 × 10^3^	3.2225 × 10^3^	3.3803 × 10^3^	3.0543 × 10^3^	3.0700 × 10^3^
	std	**3.9504 × 10^1^**	8.3022 × 10^1^	1.0842 × 10^2^	1.7300 × 10^2^	6.9108 × 10^1^	1.4389 × 10^2^	1.3651 × 10^2^	1.4758 × 10^2^	4.2744 × 10^1^	1.2192 × 10^2^	1.0384 × 10^2^	9.1536 × 10^1^
F29	mean	3.2431 × 10^3^	3.1715 × 10^3^	3.1583 × 10^3^	**3.1458 × 10^3^**	3.1622 × 10^3^	3.1505 × 10^3^	3.1780 × 10^3^	3.1507 × 10^3^	3.2023 × 10^3^	3.3319 × 10^3^	3.1510 × 10^3^	3.1341 × 10^3^
	std	5.6363 × 10^1^	2.5736 × 10^1^	1.5211 × 10^1^	1.5650 × 10^1^	3.9390 × 10^1^	**1.0004 × 10^1^**	2.3496 × 10^1^	1.2438 × 10^1^	1.9303 × 10^1^	8.0801 × 10^1^	9.2151 × 10	1.0926 × 10
F30	mean	2.0786 × 10^4^	6.2193 × 10^4^	**1.0382 × 10^4^**	8.9056 × 10^4^	2.4989 × 10^4^	2.3104 × 10^5^	2.2292 × 10^5^	6.2358 × 10^4^	2.3561 × 10^5^	1.4363 × 10^6^	1.1519 × 10^4^	3.9048 × 10^3^
	std	1.8062 × 10^4^	2.0617 × 10^5^	**5.7099 × 10^3^**	2.4803 × 10^5^	1.7666 × 10^4^	3.6582 × 10^5^	4.1565 × 10^5^	2.1568 × 10^5^	1.6920 × 10^5^	1.0987 × 10^6^	1.3166 × 10^4^	1.8510 × 10^2^

**Table 3:** Experimental results of CEC2017 (dim = 30).

ID	Metric	PSO	SO	GRO	SBOA	ED	ESC	HHWOA	IGWO	MSPBO	QOCSPBO	SPBO	MESPBO
F1	mean	2.4596 × 10^5^	4.5560 × 10^4^	1.5599 × 10^6^	6.8940 × 10^3^	2.9922 × 10^3^	3.5303 × 10^3^	4.6072 × 10^3^	3.8406 × 10^5^	3.9490 × 10^6^	4.6773 × 10^9^	1.8896 × 10^2^	**1.5596 × 10^2^**
	std	1.7503 × 10^5^	6.6804 × 10^4^	1.8106 × 10^6^	6.7951 × 10^3^	2.3195 × 10^3^	3.4202 × 10^3^	5.1698 × 10^3^	2.2382 × 10^5^	1.7832 × 10^6^	1.2942 × 10^9^	9.7710 × 10^1^	5.1543 × 10^1^
F2	mean	**1.7501 × 10^10^**	2.2070 × 10^18^	2.4786 × 10^22^	4.7204 × 10^12^	3.5066 × 10^22^	1.4068 × 10^15^	9.2087 × 10^16^	3.0351 × 10^16^	1.5720 × 10^29^	1.4922 × 10^38^	6.0646 × 10^4^	5.0359 × 10^8^
	std	**5.3363 × 10^10^**	7.6977 × 10^18^	1.2492 × 10^23^	1.0226 × 10^13^	1.3679 × 10^23^	6.7197 × 10^15^	3.7255 × 10^17^	9.0360 × 10^16^	3.4408 × 10^29^	5.1992 × 10^38^	2.4761 × 10^5^	4.9406 × 10^8^
F3	mean	5.6259 × 10^3^	5.6741 × 10^4^	3.4051 × 10^4^	6.6270 × 10^3^	8.0714 × 10^4^	4.0339 × 10^4^	**3.0000 × 10^2^**	5.7968 × 10^3^	1.3640 × 10^5^	7.3497 × 10^4^	9.1277 × 10^4^	2.0269 × 10^3^
	std	2.8127 × 10^3^	8.4409 × 10^3^	6.2035 × 10^3^	3.1070 × 10^3^	1.5312 × 10^4^	1.0266 × 10^4^	**3.9070 × 10^−3^**	3.1029 × 10^3^	2.0652 × 10^4^	8.2942 × 10^3^	1.9301 × 10^4^	3.1745 × 10^2^
F4	mean	**4.6402 × 10^2^**	5.0305 × 10^2^	5.1650 × 10^2^	4.9837 × 10^2^	4.7466 × 10^2^	5.0649 × 10^2^	4.7536 × 10^2^	4.9601 × 10^2^	5.9628 × 10^2^	1.6792 × 10^3^	4.2963 × 10^2^	4.7313 × 10^2^
	std	2.6125 × 10^1^	2.4952 × 10^1^	1.9550 × 10^1^	1.9485 × 10^1^	3.5493 × 10^1^	1.4252 × 10^1^	3.0436 × 10^1^	**1.2369 × 10^1^**	2.3736 × 10^1^	5.3309 × 10^2^	2.9474 × 10^1^	5.0237 × 10^0^
F5	mean	6.7834 × 10^2^	5.6314 × 10^2^	5.7199 × 10^2^	**5.6033 × 10^2^**	6.4726 × 10^2^	5.7734 × 10^2^	5.8799 × 10^2^	5.7573 × 10^2^	6.9789 × 10^2^	8.2933 × 10^2^	5.5268 × 10^2^	5.2816 × 10^2^
	std	2.7151 × 10^1^	1.6095 × 10^1^	1.6196 × 10^1^	1.9374 × 10^1^	1.5657 × 10^1^	1.9956 × 10^1^	2.0267 × 10^1^	4.5163 × 10^1^	**1.4828 × 10^1^**	2.7358 × 10^1^	9.2838 × 10^0^	4.4624 × 10^0^
F6	mean	6.3977 × 10^2^	6.0262 × 10^2^	6.0432 × 10^2^	6.0048 × 10^2^	6.0402 × 10^2^	**6.0000 × 10^2^**	6.0500 × 10^2^	6.0047 × 10^2^	6.0210 × 10^2^	6.7152 × 10^2^	6.0000 × 10^2^	6.0000 × 10^2^
	std	7.1212 × 10^0^	1.9415 × 10^0^	1.9594 × 10^0^	5.7791 × 10^−1^	3.9740 × 10^0^	**2.0211 × 10^−3^**	3.6091 × 10^0^	2.2918 × 10^−1^	5.6066 × 10^−1^	5.7272 × 10^0^	**8.4444 × 10^−14^**	5.7673 × 10^−4^
F7	mean	8.5063 × 10^2^	8.2078 × 10^2^	**8.0959 × 10^2^**	8.1224 × 10^2^	8.6623 × 10^2^	8.2038 × 10^2^	8.6855 × 10^2^	8.2772 × 10^2^	9.4479 × 10^2^	1.3031 × 10^3^	7.7617 × 10^2^	7.5892 × 10^2^
	std	2.7603 × 10^1^	2.9088 × 10^1^	3.3694 × 10^1^	3.1838 × 10^1^	2.1647 × 10^1^	1.4310 × 10^1^	4.5764 × 10^1^	5.8842 × 10^1^	**1.1883 × 10^1^**	6.8417 × 10^1^	7.0361 × 10^0^	3.5724 × 10^0^
F8	mean	9.2021 × 10^2^	**8.5703 × 10^2^**	8.7075 × 10^2^	8.6112 × 10^2^	9.3687 × 10^2^	8.7087 × 10^2^	8.7879 × 10^2^	8.6354 × 10^2^	9.9632 × 10^2^	1.0348 × 10^3^	8.5800 × 10^2^	8.3075 × 10^2^
	std	1.6790 × 10^1^	**1.0402 × 10^1^**	1.6366 × 10^1^	2.0792 × 10^1^	1.9235 × 10^1^	1.7022 × 10^1^	2.2166 × 10^1^	4.4014 × 10^1^	1.4977 × 10^1^	2.1772 × 10^1^	9.9026 × 10^0^	4.5211 × 10^0^
F9	mean	4.7558 × 10^3^	1.2920 × 10^3^	1.1921 × 10^3^	9.6899 × 10^2^	1.2481 × 10^3^	**9.0026 × 10^2^**	1.3374 × 10^3^	9.0984 × 10^2^	1.3245 × 10^3^	8.1339 × 10^3^	1.0401 × 10^3^	9.0022 × 10^2^
	std	1.3851 × 10^3^	2.8686 × 10^2^	2.6363 × 10^2^	8.9781 × 10^1^	2.6585 × 10^2^	**4.6554 × 10^−1^**	3.2112 × 10^2^	2.7461 × 10^1^	1.8158 × 10^2^	8.1707 × 10^2^	8.7559 × 10^1^	1.4123 × 10^−1^
F10	mean	4.7756 × 10^3^	**3.6220 × 10^3^**	4.2873 × 10^3^	4.0910 × 10^3^	5.0743 × 10^3^	6.5289 × 10^3^	4.7189 × 10^3^	6.4259 × 10^3^	8.4369 × 10^3^	7.3814 × 10^3^	2.9533 × 10^3^	3.5077 × 10^3^
	std	7.3609 × 10^2^	1.1449 × 10^3^	5.8269 × 10^2^	6.3615 × 10^2^	**2.9302 × 10^2^**	4.7613 × 10^2^	6.5698 × 10^2^	2.2447 × 10^3^	3.1474 × 10^2^	5.5562 × 10^2^	2.0419 × 10^2^	2.9656 × 10^2^
F11	mean	1.2083 × 10^3^	1.2477 × 10^3^	1.2030 × 10^3^	**1.1668 × 10^3^**	1.1935 × 10^3^	1.1767 × 10^3^	1.1823 × 10^3^	1.1858 × 10^3^	1.5164 × 10^3^	2.6927 × 10^3^	1.2307 × 10^3^	1.1153 × 10^3^
	std	2.6490 × 10^1^	4.9339 × 10^1^	3.1518 × 10^1^	3.0609 × 10^1^	3.9320 × 10^1^	**2.0054 × 10^1^**	3.6721 × 10^1^	2.7607 × 10^1^	8.6856 × 10^1^	5.3808 × 10^2^	6.4523 × 10^1^	4.0984 × 10^0^
F12	mean	1.5396 × 10^6^	7.2948 × 10^5^	7.7828 × 10^5^	3.5819 × 10^5^	3.5131 × 10^5^	7.2829 × 10^5^	**6.1237 × 10^4^**	1.4945 × 10^6^	3.5254 × 10^6^	7.5238 × 10^8^	3.1685 × 10^5^	4.0537 × 10^4^
	std	1.3335 × 10^6^	8.1913 × 10^5^	6.1627 × 10^5^	2.8788 × 10^5^	2.9175 × 10^5^	6.3280 × 10^5^	**7.1867 × 10^4^**	9.2758 × 10^5^	1.5726 × 10^6^	3.7548 × 10^8^	2.2746 × 10^5^	1.1669 × 10^4^
F13	mean	**1.4813 × 10^4^**	2.0626 × 10^4^	2.0060 × 10^4^	2.2367 × 10^4^	2.7550 × 10^4^	1.6355 × 10^4^	1.9835 × 10^4^	1.4977 × 10^5^	2.0470 × 10^4^	4.9138 × 10^7^	6.7450 × 10^3^	2.7933 × 10^3^
	std	1.7632 × 10^4^	1.6147 × 10^4^	1.4572 × 10^4^	1.9108 × 10^4^	1.9773 × 10^4^	1.1804 × 10^4^	1.8113 × 10^4^	8.5169 × 10^4^	**1.0036 × 10^4^**	3.7143 × 10^7^	4.8294 × 10^3^	7.1352 × 10^2^
F14	mean	1.5928 × 10^4^	2.9210 × 10^4^	1.2283 × 10^4^	1.6767 × 10^4^	3.4752 × 10^4^	3.7845 × 10^4^	**1.4855 × 10^3^**	7.9845 × 10^3^	2.0494 × 10^5^	1.3408 × 10^6^	5.6801 × 10^4^	2.4274 × 10^3^
	std	2.5451 × 10^4^	3.9078 × 10^4^	9.4987 × 10^3^	1.9670 × 10^4^	3.6214 × 10^4^	4.8333 × 10^4^	**6.0844 × 10^1^**	6.2977 × 10^3^	1.9066 × 10^5^	1.2779 × 10^6^	3.9885 × 10^4^	4.3621 × 10^2^
F15	mean	8.3592 × 10^3^	7.5755 × 10^3^	6.8856 × 10^3^	8.9023 × 10^3^	6.2047 × 10^3^	5.0609 × 10^3^	**1.6050 × 10^3^**	2.2718 × 10^4^	4.6087 × 10^3^	1.3321 × 10^6^	2.0984 × 10^3^	2.5486 × 10^3^
	std	9.8873 × 10^3^	7.0157 × 10^3^	5.3948 × 10^3^	8.5654 × 10^3^	4.8499 × 10^3^	4.2108 × 10^3^	**2.4807 × 10^2^**	1.7717 × 10^4^	2.6104 × 10^3^	9.6807 × 10^5^	6.7721 × 10^2^	4.8055 × 10^2^
F16	mean	2.5776 × 10^3^	2.3622 × 10^3^	2.1912 × 10^3^	2.2377 × 10^3^	2.8702 × 10^3^	**2.0357 × 10^3^**	2.5562 × 10^3^	2.1639 × 10^3^	3.2951 × 10^3^	4.0369 × 10^3^	2.1409 × 10^3^	1.8364 × 10^3^
	std	2.8817 × 10^2^	2.4040 × 10^2^	1.6478 × 10^2^	2.8943 × 10^2^	**1.6047 × 10^2^**	1.9427 × 10^2^	3.5644 × 10^2^	4.4080 × 10^2^	1.9081 × 10^2^	4.0050 × 10^2^	1.1976 × 10^2^	1.0272 × 10^2^
F17	mean	2.3070 × 10^3^	1.9907 × 10^3^	1.8437 × 10^3^	1.8421 × 10^3^	2.1334 × 10^3^	**1.8305 × 10^3^**	2.1745 × 10^3^	1.8507 × 10^3^	2.3505 × 10^3^	2.6310 × 10^3^	1.8361 × 10^3^	1.8012 × 10^3^
	std	2.1975 × 10^2^	1.6693 × 10^2^	**5.9343 × 10^1^**	8.4069 × 10^1^	1.2962 × 10^2^	9.3385 × 10^1^	1.7479 × 10^2^	1.1620 × 10^2^	1.4710 × 10^2^	2.3809 × 10^2^	6.0487 × 10^1^	1.8146 × 10^1^
F18	mean	3.6281 × 10^5^	4.0398 × 10^5^	3.1386 × 10^5^	3.0627 × 10^5^	7.0699 × 10^5^	4.9221 × 10^5^	**9.4064 × 10^3^**	1.4821 × 10^5^	3.2111 × 10^6^	7.2249 × 10^6^	1.2175 × 10^5^	7.6168 × 10^4^
	std	2.7583 × 10^5^	3.1315 × 10^5^	2.2033 × 10^5^	2.5376 × 10^5^	3.5569 × 10^5^	5.3267 × 10^5^	**1.1557 × 10^4^**	8.8047 × 10^4^	1.5455 × 10^6^	6.1290 × 10^6^	6.1649 × 10^4^	2.3466 × 10^4^
F19	mean	1.1334 × 10^4^	1.0154 × 10^4^	7.9412 × 10^3^	1.1190 × 10^4^	9.4179 × 10^3^	6.1317 × 10^3^	**5.6914 × 10^3^**	1.5536 × 10^4^	8.3464 × 10^3^	1.0231 × 10^7^	2.9665 × 10^3^	2.2927 × 10^3^
	std	1.0137 × 10^4^	8.8497 × 10^3^	6.2186 × 10^3^	1.0766 × 10^4^	1.2030 × 10^4^	6.8140 × 10^3^	1.3776 × 10^4^	1.5239 × 10^4^	**5.1228 × 10^3^**	7.4936 × 10^6^	1.1357 × 10^3^	1.9748 × 10^2^
F20	mean	2.6406 × 10^3^	2.3675 × 10^3^	2.2448 × 10^3^	2.1960 × 10^3^	2.5458 × 10^3^	2.1610 × 10^3^	2.4907 × 10^3^	**2.1540 × 10^3^**	2.6468 × 10^3^	2.7801 × 10^3^	2.1671 × 10^3^	2.0962 × 10^3^
	std	2.1483 × 10^2^	1.4741 × 10^2^	1.0063 × 10^2^	1.0229 × 10^2^	1.1028 × 10^2^	1.1205 × 10^2^	1.8798 × 10^2^	**7.8455 × 10^1^**	1.7222 × 10^2^	1.7913 × 10^2^	6.2669 × 10^1^	2.3384 × 10^1^
F21	mean	2.4736 × 10^3^	2.3630 × 10^3^	2.3575 × 10^3^	**2.3479 × 10^3^**	2.4452 × 10^3^	2.3663 × 10^3^	2.3867 × 10^3^	2.3600 × 10^3^	2.4948 × 10^3^	2.6302 × 10^3^	2.3462 × 10^3^	2.3294 × 10^3^
	std	6.1024 × 10^1^	**1.1820 × 10^1^**	1.6702 × 10^1^	1.4040 × 10^1^	1.7554 × 10^1^	2.0893 × 10^1^	2.8716 × 10^1^	4.0240 × 10^1^	1.4360 × 10^1^	3.6998 × 10^1^	4.3418 × 10^1^	4.9411 × 10^0^
F22	mean	4.7374 × 10^3^	3.2874 × 10^3^	2.3102 × 10^3^	**2.3006 × 10^3^**	5.5191 × 10^3^	2.8079 × 10^3^	3.9133 × 10^3^	2.9150 × 10^3^	7.7309 × 10^3^	5.4541 × 10^3^	2.3821 × 10^3^	2.3000 × 10^3^
	std	2.2074 × 10^3^	1.2087 × 10^3^	4.7894 × 10^0^	**1.1814 × 10^0^**	1.8152 × 10^3^	1.5678 × 10^3^	2.2077 × 10^3^	1.8695 × 10^3^	2.3602 × 10^3^	1.8640 × 10^3^	4.4658 × 10^2^	4.5485 × 10^−7^
F23	mean	3.0755 × 10^3^	2.7434 × 10^3^	2.7174 × 10^3^	2.7017 × 10^3^	2.8089 × 10^3^	**2.6986 × 10^3^**	2.7675 × 10^3^	2.7060 × 10^3^	2.8412 × 10^3^	3.2794 × 10^3^	2.7113 × 10^3^	2.6900 × 10^3^
	std	1.2654 × 10^2^	2.0901 × 10^1^	1.8198 × 10^1^	1.3847 × 10^1^	2.0016 × 10^1^	2.0133 × 10^1^	3.0742 × 10^1^	4.0844 × 10^1^	**1.3533 × 10^1^**	1.2719 × 10^2^	1.0862 × 10^1^	7.1280 × 10^0^
F24	mean	3.1811 × 10^3^	2.8933 × 10^3^	2.8786 × 10^3^	**2.8666 × 10^3^**	2.9771 × 10^3^	2.9091 × 10^3^	2.9258 × 10^3^	2.9040 × 10^3^	3.0199 × 10^3^	3.3914 × 10^3^	2.8743 × 10^3^	2.8589 × 10^3^
	std	8.8045 × 10^1^	1.4178 × 10^1^	1.5979 × 10^1^	1.7443 × 10^1^	2.7855 × 10^1^	1.4530 × 10^1^	4.3870 × 10^1^	6.3692 × 10^1^	**1.1416 × 10^1^**	1.2368 × 10^2^	1.5190 × 10^2^	5.0005 × 10^0^
F25	mean	**2.8845 × 10^3^**	2.8932 × 10^3^	2.9117 × 10^3^	2.8980 × 10^3^	2.8904 × 10^3^	2.8891 × 10^3^	2.8981 × 10^3^	2.8872 × 10^3^	2.9390 × 10^3^	3.2345 × 10^3^	2.8838 × 10^3^	2.8848 × 10^3^
	std	1.3505 × 10^1^	9.8600 × 10^0^	1.9462 × 10^1^	1.7328 × 10^1^	7.9075 × 10^0^	4.4094 × 10^0^	1.6850 × 10^1^	**1.6754 × 10^0^**	1.0540 × 10^1^	8.7998 × 10^1^	1.8792 × 10^−1^	1.5740 × 10^0^
F26	mean	5.7774 × 10^3^	4.7504 × 10^3^	**3.6235 × 10^3^**	3.8717 × 10^3^	5.0612 × 10^3^	4.0406 × 10^3^	5.0263 × 10^3^	3.8676 × 10^3^	5.6073 × 10^3^	7.9669 × 10^3^	3.0703 × 10^3^	3.7417 × 10^3^
	std	1.9820 × 10^3^	2.6490 × 10^2^	7.2199 × 10^2^	5.9975 × 10^2^	6.5764 × 10^2^	2.3494 × 10^2^	4.0932 × 10^2^	4.7102 × 10^2^	**1.4298 × 10^2^**	1.3764 × 10^3^	5.6179 × 10^2^	4.9138 × 10^2^
F27	mean	3.3211 × 10^3^	3.2571 × 10^3^	3.2398 × 10^3^	3.2130 × 10^3^	3.2406 × 10^3^	3.2121 × 10^3^	3.2461 × 10^3^	**3.2021 × 10^3^**	3.2433 × 10^3^	3.6311 × 10^3^	3.2112 × 10^3^	3.1971 × 10^3^
	std	1.9337 × 10^2^	1.9526 × 10^1^	1.1989 × 10^1^	8.8266 × 10^0^	1.8051 × 10^1^	**6.9371 × 10^0^**	2.8123 × 10^1^	1.0024 × 10^1^	9.0040 × 10^0^	1.5912 × 10^2^	4.6880 × 10^0^	4.6605 × 10^0^
F28	mean	3.2370 × 10^3^	3.2646 × 10^3^	3.2669 × 10^3^	3.2150 × 10^3^	3.2334 × 10^3^	3.2322 × 10^3^	**3.1649 × 10^3^**	3.2194 × 10^3^	3.3506 × 10^3^	4.0120 × 10^3^	3.1591 × 10^3^	3.1891 × 10^3^
	std	2.3163 × 10^1^	2.1089 × 10^1^	2.6497 × 10^1^	1.2274 × 10^1^	2.4596 × 10^1^	1.6115 × 10^1^	6.3881 × 10^1^	**1.0705 × 10^1^**	2.1026 × 10^1^	2.4593 × 10^2^	3.9725 × 10^1^	2.3434 × 10^1^
F29	mean	4.1611 × 10^3^	3.8186 × 10^3^	3.6129 × 10^3^	3.5210 × 10^3^	3.7897 × 10^3^	3.4786 × 10^3^	3.8356 × 10^3^	**3.4676 × 10^3^**	4.3602 × 10^3^	5.3447 × 10^3^	3.4467 × 10^3^	3.4540 × 10^3^
	std	2.0489 × 10^2^	1.8372 × 10^2^	1.4201 × 10^2^	1.5489 × 10^2^	1.2275 × 10^2^	**9.1715 × 10^1^**	2.4729 × 10^2^	9.2550 × 10^1^	1.7632 × 10^2^	4.6063 × 10^2^	6.8469 × 10^1^	3.4203 × 10^1^
F30	mean	4.4794 × 10^4^	2.3836 × 10^4^	1.6167 × 10^4^	1.2819 × 10^4^	4.1836 × 10^4^	1.0345 × 10^4^	**9.6933 × 10^3^**	2.1012 × 10^5^	2.2835 × 10^5^	7.3960 × 10^7^	6.5580 × 10^3^	8.7688 × 10^3^
	std	2.5129 × 10^4^	3.9028 × 10^4^	8.2884 × 10^3^	4.6478 × 10^3^	5.4515 × 10^4^	**3.3683 × 10^3^**	3.9358 × 10^3^	1.4625 × 10^5^	1.1340 × 10^5^	7.6312 × 10^7^	6.0648 × 10^2^	1.2881 × 10^3^

**Table 4:** Experimental results of CEC2017 (dim = 50).

ID	Metric	PSO	SO	GRO	SBOA	ED	ESC	HHWOA	IGWO	MSPBO	QOCSPBO	SPBO	MESPBO
F1	mean	8.6734 × 10^6^	2.3685 × 10^6^	5.3913 × 10^8^	1.2142 × 10^4^	3.3006 × 10^4^	2.7644 × 10^3^	3.9216 × 10^3^	1.2373 × 10^7^	6.9607 × 10^8^	2.3090 × 10^10^	8.5150 × 10^2^	**3.8769 × 10^2^**
	std	3.4959 × 10^6^	1.5953 × 10^6^	3.4521 × 10^8^	1.1842 × 10^4^	3.8494 × 10^4^	2.7582 × 10^3^	4.8496 × 10^3^	5.8869 × 10^6^	2.1505 × 10^8^	4.9392 × 10^9^	8.9134 × 10^2^	1.3170 × 10^2^
F2	mean	**1.1321 × 10^23^**	7.7286 × 10^41^	1.2534 × 10^47^	1.8707 × 10^33^	1.1498 × 10^44^	1.0850 × 10^42^	1.5421 × 10^42^	5.7637 × 10^37^	2.4870 × 10^61^	4.1030 × 10^73^	2.4739 × 10^13^	7.4016 × 10^26^
	std	**2.3415 × 10^23^**	1.8387 × 10^42^	4.9974 × 10^47^	1.0239 × 10^34^	5.9854 × 10^44^	3.9538 × 10^42^	6.2931 × 10^42^	2.1849 × 10^38^	7.2696 × 10^61^	2.2083 × 10^74^	1.3437 × 10^14^	1.0605 × 10^27^
F3	mean	8.0519 × 10^4^	1.3709 × 10^5^	1.0701 × 10^5^	4.2937 × 10^4^	2.4239 × 10^5^	1.5926 × 10^5^	**1.4391 × 10^3^**	3.1961 × 10^4^	3.1827 × 10^5^	1.7614 × 10^5^	2.4043 × 10^5^	4.2018 × 10^4^
	std	1.9113 × 10^4^	1.4660 × 10^4^	1.8568 × 10^4^	1.0579 × 10^4^	3.4754 × 10^4^	2.7322 × 10^4^	**1.2826 × 10^3^**	7.2848 × 10^3^	3.9136 × 10^4^	1.4209 × 10^4^	2.5516 × 10^4^	3.7041 × 10^3^
F4	mean	5.3900 × 10^2^	6.0547 × 10^2^	7.4178 × 10^2^	5.4650 × 10^2^	5.5164 × 10^2^	5.9117 × 10^2^	**5.2955 × 10^2^**	5.9929 × 10^2^	9.2187 × 10^2^	4.9451 × 10^3^	4.2880 × 10^2^	4.8216 × 10^2^
	std	4.7706 × 10^1^	5.3270 × 10^1^	9.6985 × 10^1^	5.7918 × 10^1^	**3.9988 × 10^1^**	4.1892 × 10^1^	4.7105 × 10^1^	4.6263 × 10^1^	5.9182 × 10^1^	1.4072 × 10^3^	1.5886 × 10^1^	2.6648 × 10^1^
F5	mean	7.7107 × 10^2^	**6.3086 × 10^2^**	6.9511 × 10^2^	6.7350 × 10^2^	8.4367 × 10^2^	6.9753 × 10^2^	6.9542 × 10^2^	6.4328 × 10^2^	9.2641 × 10^2^	1.0411 × 10^3^	6.3653 × 10^2^	5.6668 × 10^2^
	std	3.1775 × 10^1^	2.2643 × 10^1^	3.3310 × 10^1^	3.4169 × 10^1^	2.3217 × 10^1^	3.6683 × 10^1^	3.5800 × 10^1^	4.8374 × 10^1^	**2.1667 × 10^1^**	3.1057 × 10^1^	1.3004 × 10^1^	5.5336 × 10^0^
F6	mean	6.5205 × 10^2^	6.1019 × 10^2^	6.1627 × 10^2^	6.0634 × 10^2^	6.2313 × 10^2^	**6.0010 × 10^2^**	6.1961 × 10^2^	6.0213 × 10^2^	6.1160 × 10^2^	6.8998 × 10^2^	6.0000 × 10^2^	6.0015 × 10^2^
	std	6.0171 × 10^0^	5.2724 × 10^0^	4.6810 × 10^0^	3.7230 × 10^0^	5.5937 × 10^0^	**1.1072 × 10^−1^**	7.0140 × 10^0^	9.1144 × 10^−1^	2.1198 × 10^0^	6.4049 × 10^0^	**7.3131 × 10^−14^**	3.6565 × 10^−2^
F7	mean	1.0737 × 10^3^	9.4840 × 10^2^	1.0219 × 10^3^	9.8636 × 10^2^	1.1433 × 10^3^	9.7989 × 10^2^	1.1169 × 10^3^	**9.4569 × 10^2^**	1.2161 × 10^3^	1.9081 × 10^3^	8.5993 × 10^2^	8.1874 × 10^2^
	std	6.2901 × 10^1^	3.6518 × 10^1^	6.2968 × 10^1^	6.8683 × 10^1^	5.4777 × 10^1^	2.6986 × 10^1^	7.8483 × 10^1^	7.9035 × 10^1^	**2.0705 × 10^1^**	8.4598 × 10^1^	1.2753 × 10^1^	9.5937 × 10^0^
F8	mean	1.0985 × 10^3^	**9.2707 × 10^2^**	1.0027 × 10^3^	9.5902 × 10^2^	1.1365 × 10^3^	9.7553 × 10^2^	9.9297 × 10^2^	9.7436 × 10^2^	1.2307 × 10^3^	1.3336 × 10^3^	9.3542 × 10^2^	8.6486 × 10^2^
	std	2.9356 × 10^1^	**1.7435 × 10^1^**	3.9263 × 10^1^	3.2953 × 10^1^	2.4816 × 10^1^	4.8029 × 10^1^	4.1708 × 10^1^	9.1353 × 10^1^	2.0802 × 10^1^	3.4417 × 10^1^	1.5205 × 10^1^	6.3932 × 10^0^
F9	mean	2.2509 × 10^4^	2.4686 × 10^3^	4.0613 × 10^3^	2.9595 × 10^3^	1.0470 × 10^4^	**9.6268 × 10^2^**	3.1924 × 10^3^	1.5135 × 10^3^	6.1152 × 10^3^	2.9383 × 10^4^	1.8765 × 10^3^	9.1989 × 10^2^
	std	5.2617 × 10^3^	8.7294 × 10^2^	1.1291 × 10^3^	1.2072 × 10^3^	3.7003 × 10^3^	**5.8707 × 10^1^**	1.0576 × 10^3^	6.6138 × 10^2^	1.2177 × 10^3^	2.8397 × 10^3^	3.0329 × 10^2^	6.1594 × 10^0^
F10	mean	7.3854 × 10^3^	9.0578 × 10^3^	7.3818 × 10^3^	**6.6217 × 10^3^**	8.8334 × 10^3^	1.2089 × 10^4^	7.8758 × 10^3^	1.2131 × 10^4^	1.5200 × 10^4^	1.2632 × 10^4^	4.7369 × 10^3^	6.5693 × 10^3^
	std	9.4706 × 10^2^	2.8790 × 10^3^	7.8832 × 10^2^	9.3155 × 10^2^	4.1864 × 10^2^	6.9525 × 10^2^	9.7975 × 10^2^	3.5238 × 10^3^	**3.4271 × 10^2^**	6.1356 × 10^2^	2.3504 × 10^2^	5.1587 × 10^2^
F11	mean	1.3285 × 10^3^	1.5532 × 10^3^	2.0524 × 10^3^	**1.2637 × 10^3^**	1.5698 × 10^3^	1.3574 × 10^3^	1.3268 × 10^3^	1.4355 × 10^3^	5.7594 × 10^3^	7.5358 × 10^3^	1.7598 × 10^3^	1.2226 × 10^3^
	std	4.4182 × 10^1^	1.2958 × 10^2^	4.7057 × 10^2^	**4.3111 × 10^1^**	1.5205 × 10^2^	1.3242 × 10^2^	6.4318 × 10^1^	6.8734 × 10^1^	1.2641 × 10^3^	1.3115 × 10^3^	4.8893 × 10^2^	1.4840 × 10^1^
F12	mean	1.1469 × 10^7^	1.1554 × 10^7^	1.3915 × 10^7^	4.3907 × 10^6^	4.3492 × 10^6^	5.0536 × 10^6^	**6.4706 × 10^5^**	2.1394 × 10^7^	1.5508 × 10^8^	5.4347 × 10^9^	1.6127 × 10^6^	4.8023 × 10^5^
	std	5.9075 × 10^6^	8.2347 × 10^6^	1.0430 × 10^7^	2.3229 × 10^6^	3.0015 × 10^6^	3.2520 × 10^6^	**4.1159 × 10^5^**	1.0725 × 10^7^	4.2305 × 10^7^	1.8336 × 10^9^	5.6886 × 10^5^	1.6396 × 10^5^
F13	mean	2.9101 × 10^4^	3.8254 × 10^4^	9.1108 × 10^3^	1.1536 × 10^4^	**8.1246 × 10^3^**	9.4035 × 10^3^	1.1992 × 10^4^	3.6843 × 10^5^	1.8224 × 10^5^	7.6275 × 10^8^	3.2039 × 10^3^	2.3976 × 10^3^
	std	1.1205 × 10^4^	2.3960 × 10^4^	**3.6171 × 10^3^**	9.0202 × 10^3^	6.8628 × 10^3^	4.1045 × 10^3^	9.3041 × 10^3^	2.0270 × 10^5^	1.3935 × 10^5^	4.2349 × 10^8^	2.4444 × 10^3^	6.2663 × 10^2^
F14	mean	1.3427 × 10^5^	1.8915 × 10^5^	1.2733 × 10^5^	1.7565 × 10^5^	5.0495 × 10^5^	3.7153 × 10^5^	**9.1018 × 10^3^**	8.6698 × 10^4^	1.6267 × 10^6^	8.0739 × 10^6^	6.0671 × 10^5^	2.0301 × 10^4^
	std	8.7992 × 10^4^	1.3875 × 10^5^	8.9269 × 10^4^	1.4661 × 10^5^	3.3528 × 10^5^	4.2982 × 10^5^	**8.0683 × 10^3^**	6.9063 × 10^4^	6.0420 × 10^5^	8.8328 × 10^6^	4.2494 × 10^5^	7.2294 × 10^3^
F15	mean	1.0373 × 10^4^	1.2367 × 10^4^	9.1041 × 10^3^	1.1467 × 10^4^	**6.9715 × 10^3^**	7.6237 × 10^3^	1.0527 × 10^4^	1.0932 × 10^5^	2.5429 × 10^4^	6.8868 × 10^7^	4.1130 × 10^3^	2.4394 × 10^3^
	std	7.2767 × 10^3^	5.8638 × 10^3^	4.7893 × 10^3^	7.0539 × 10^3^	6.4609 × 10^3^	**4.7378 × 10^3^**	8.9865 × 10^3^	8.0293 × 10^4^	1.2912 × 10^4^	6.5183 × 10^7^	2.6936 × 10^3^	3.8271 × 10^2^
F16	mean	3.2325 × 10^3^	2.9354 × 10^3^	2.8243 × 10^3^	2.7223 × 10^3^	4.0200 × 10^3^	3.0385 × 10^3^	3.2836 × 10^3^	**2.5526 × 10^3^**	5.0559 × 10^3^	6.4076 × 10^3^	2.7871 × 10^3^	2.5329 × 10^3^
	std	4.7497 × 10^2^	3.6022 × 10^2^	**2.9499 × 10^2^**	3.6445 × 10^2^	3.2554 × 10^2^	3.6073 × 10^2^	4.1614 × 10^2^	4.4912 × 10^2^	3.0523 × 10^2^	8.6449 × 10^2^	2.2885 × 10^2^	2.0594 × 10^2^
F17	mean	3.1660 × 10^3^	2.7204 × 10^3^	2.6991 × 10^3^	2.5763 × 10^3^	3.3163 × 10^3^	**2.5442 × 10^3^**	3.1080 × 10^3^	2.5811 × 10^3^	3.9918 × 10^3^	4.4062 × 10^3^	2.4494 × 10^3^	2.4128 × 10^3^
	std	2.3482 × 10^2^	2.6615 × 10^2^	2.5654 × 10^2^	2.9932 × 10^2^	2.4172 × 10^2^	2.4353 × 10^2^	3.2673 × 10^2^	4.1630 × 10^2^	**2.0232 × 10^2^**	6.4877 × 10^2^	1.5883 × 10^2^	1.6441 × 10^2^
F18	mean	1.5490 × 10^6^	2.1561 × 10^6^	1.5097 × 10^6^	1.1952 × 10^6^	4.3640 × 10^6^	3.5759 × 10^6^	**4.1173 × 10^4^**	6.0097 × 10^5^	2.1350 × 10^7^	3.8823 × 10^7^	6.2119 × 10^5^	3.2767 × 10^5^
	std	8.9012 × 10^5^	1.3394 × 10^6^	1.2341 × 10^6^	7.4304 × 10^5^	2.6864 × 10^6^	2.7580 × 10^6^	**3.1312 × 10^4^**	4.0373 × 10^5^	8.2142 × 10^6^	2.7541 × 10^7^	3.4535 × 10^5^	7.4511 × 10^4^
F19	mean	1.6640 × 10^4^	1.7242 × 10^4^	1.7670 × 10^4^	1.8365 × 10^4^	**1.1502 × 10^4^**	1.6405 × 10^4^	1.8139 × 10^4^	6.2506 × 10^4^	2.1137 × 10^4^	2.1800 × 10^7^	5.2383 × 10^3^	2.3734 × 10^3^
	std	8.2542 × 10^3^	1.2242 × 10^4^	1.0550 × 10^4^	1.1407 × 10^4^	1.0138 × 10^4^	9.5165 × 10^3^	1.2900 × 10^4^	3.1326 × 10^4^	**5.9293 × 10^3^**	1.8551 × 10^7^	2.6114 × 10^3^	3.6538 × 10^2^
F20	mean	3.1222 × 10^3^	2.9760 × 10^3^	2.6872 × 10^3^	**2.6268 × 10^3^**	3.5373 × 10^3^	2.7731 × 10^3^	3.0128 × 10^3^	2.9690 × 10^3^	4.1382 × 10^3^	3.6252 × 10^3^	2.6225 × 10^3^	2.5935 × 10^3^
	std	2.8785 × 10^2^	4.1701 × 10^2^	2.4773 × 10^2^	2.4663 × 10^2^	**1.1407 × 10^2^**	1.8729 × 10^2^	3.3030 × 10^2^	6.2917 × 10^2^	1.5947 × 10^2^	2.4593 × 10^2^	1.4247 × 10^2^	1.1464 × 10^2^
F21	mean	2.6546 × 10^3^	2.4354 × 10^3^	2.4782 × 10^3^	**2.4320 × 10^3^**	2.6341 × 10^3^	2.4840 × 10^3^	2.4960 × 10^3^	2.4514 × 10^3^	2.7096 × 10^3^	2.9960 × 10^3^	2.4481 × 10^3^	2.3720 × 10^3^
	std	4.3705 × 10^1^	2.4362 × 10^1^	3.0549 × 10^1^	2.9321 × 10^1^	2.7099 × 10^1^	4.6545 × 10^1^	4.2499 × 10^1^	9.2199 × 10^1^	**1.9422 × 10^1^**	7.3970 × 10^1^	1.8129 × 10^1^	8.4225 × 10^0^
F22	mean	9.3375 × 10^3^	1.1584 × 10^4^	**6.8459 × 10^3^**	7.3787 × 10^3^	1.0977 × 10^4^	1.3503 × 10^4^	9.7794 × 10^3^	1.3032 × 10^4^	1.6783 × 10^4^	1.4541 × 10^4^	6.1198 × 10^3^	7.5317 × 10^3^
	std	1.1614 × 10^3^	2.6307 × 10^3^	3.1716 × 10^3^	2.4111 × 10^3^	1.7210 × 10^3^	6.0105 × 10^2^	8.8270 × 10^2^	3.7830 × 10^3^	**3.3514 × 10^2^**	8.0271 × 10^2^	1.5457 × 10^3^	1.4445 × 10^3^
F23	mean	3.5836 × 10^3^	2.9259 × 10^3^	2.9283 × 10^3^	2.8593 × 10^3^	3.1107 × 10^3^	2.8762 × 10^3^	3.0062 × 10^3^	**2.8584 × 10^3^**	3.1461 × 10^3^	3.9310 × 10^3^	2.8909 × 10^3^	2.8289 × 10^3^
	std	1.2674 × 10^2^	2.9619 × 10^1^	4.1576 × 10^1^	3.3266 × 10^1^	4.9950 × 10^1^	5.2826 × 10^1^	6.5735 × 10^1^	6.8289 × 10^1^	**1.6807 × 10^1^**	1.6572 × 10^2^	2.1512 × 10^1^	1.4111 × 10^1^
F24	mean	3.5913 × 10^3^	3.0656 × 10^3^	3.0762 × 10^3^	**3.0285 × 10^3^**	3.3161 × 10^3^	3.1162 × 10^3^	3.1666 × 10^3^	3.0353 × 10^3^	3.3121 × 10^3^	4.0659 × 10^3^	3.2504 × 10^3^	3.0033 × 10^3^
	std	1.4742 × 10^2^	3.3062 × 10^1^	3.7799 × 10^1^	3.0310 × 10^1^	6.4662 × 10^1^	3.0405 × 10^1^	6.2404 × 10^1^	9.1306 × 10^1^	**1.9104 × 10^1^**	1.7734 × 10^2^	4.0101 × 10^1^	1.3501 × 10^1^
F25	mean	**2.9909 × 10^3^**	3.0948 × 10^3^	3.2390 × 10^3^	3.0895 × 10^3^	3.0797 × 10^3^	3.1086 × 10^3^	3.0535 × 10^3^	3.0938 × 10^3^	3.4498 × 10^3^	5.9306 × 10^3^	2.9687 × 10^3^	3.0183 × 10^3^
	std	4.3426 × 10^1^	3.4516 × 10^1^	5.6194 × 10^1^	3.2142 × 10^1^	2.9337 × 10^1^	**2.7134 × 10^1^**	3.8784 × 10^1^	3.3696 × 10^1^	7.4813 × 10^1^	6.3242 × 10^2^	2.0415 × 10^1^	1.6804 × 10^1^
F26	mean	8.0375 × 10^3^	6.0354 × 10^3^	5.5124 × 10^3^	5.5096 × 10^3^	7.1895 × 10^3^	**4.9611 × 10^3^**	6.7682 × 10^3^	5.4370 × 10^3^	7.9446 × 10^3^	1.3537 × 10^4^	4.0868 × 10^3^	4.6816 × 10^3^
	std	3.7319 × 10^3^	4.0146 × 10^2^	1.3363 × 10^3^	1.7288 × 10^3^	3.5661 × 10^2^	4.2283 × 10^2^	7.8138 × 10^2^	7.5542 × 10^2^	**2.1503 × 10^2^**	1.3829 × 10^3^	1.2892 × 10^3^	1.2812 × 10^2^
F27	mean	4.0311 × 10^3^	3.5952 × 10^3^	3.5472 × 10^3^	3.3197 × 10^3^	3.7637 × 10^3^	3.4086 × 10^3^	3.6112 × 10^3^	**3.3001 × 10^3^**	3.7857 × 10^3^	4.8827 × 10^3^	3.3392 × 10^3^	3.2561 × 10^3^
	std	7.5936 × 10^2^	7.7932 × 10^1^	7.1038 × 10^1^	5.4548 × 10^1^	1.3402 × 10^2^	6.0966 × 10^1^	1.7404 × 10^2^	**5.4462 × 10^1^**	8.8730 × 10^1^	7.2274 × 10^2^	2.6623 × 10^1^	1.1242 × 10^1^
F28	mean	**3.2967 × 10^3^**	3.4429 × 10^3^	3.6382 × 10^3^	3.3622 × 10^3^	3.3717 × 10^3^	3.4681 × 10^3^	3.3154 × 10^3^	3.3646 × 10^3^	4.4836 × 10^3^	6.0265 × 10^3^	3.2636 × 10^3^	3.2836 × 10^3^
	std	2.9886 × 10^1^	5.2514 × 10^1^	1.0682 × 10^2^	4.1064 × 10^1^	3.6633 × 10^1^	6.7595 × 10^1^	**2.6669 × 10^1^**	5.8427 × 10^1^	2.1107 × 10^2^	6.1755 × 10^2^	8.4268 × 10^0^	1.2148 × 10^1^
F29	mean	4.9707 × 10^3^	4.3162 × 10^3^	4.1629 × 10^3^	3.8195 × 10^3^	4.8113 × 10^3^	**3.6392 × 10^3^**	4.7845 × 10^3^	3.8262 × 10^3^	5.7029 × 10^3^	1.0002 × 10^4^	3.7594 × 10^3^	3.7718 × 10^3^
	std	4.2478 × 10^2^	2.5397 × 10^2^	2.6689 × 10^2^	2.7194 × 10^2^	4.2288 × 10^2^	**1.7120 × 10^2^**	3.4330 × 10^2^	2.5904 × 10^2^	2.1802 × 10^2^	1.6476 × 10^3^	1.2522 × 10^2^	9.8391 × 10^1^
F30	mean	3.8617 × 10^6^	2.7456 × 10^6^	1.5263 × 10^6^	**1.0568 × 10^6^**	2.9302 × 10^6^	1.2185 × 10^6^	1.0653 × 10^6^	8.8823 × 10^6^	1.1536 × 10^7^	3.7356 × 10^8^	6.5996 × 10^5^	9.2349 × 10^5^
	std	1.6101 × 10^6^	9.2617 × 10^5^	**2.8278 × 10^5^**	3.4573 × 10^5^	1.0841 × 10^6^	3.7175 × 10^5^	3.7166 × 10^5^	3.7392 × 10^6^	4.1323 × 10^6^	1.1042 × 10^8^	4.3430 × 10^4^	1.1365 × 10^5^

**Table 5:** Friedman mean rank test result.

Suites	CEC2017					
Dimensions	10		30		50	
Algorithms	M.R	T.R	M.R	T.R	M.R	T.R
PSO	9.23	10	7.77	9	7.30	9
SO	8.20	9	7.10	8	6.47	7
GRO	5.63	5	6.60	7	6.73	8
SBOA	4.67	3	4.67	3	4.50	3
ED	6.17	7	7.97	10	7.93	10
ESC	5.23	4	5.03	4	6.03	6
HHWOA	5.90	6	5.83	6	5.83	5
IGWO	6.20	8	5.53	5	5.70	4
MSPBO	9.43	11	10.57	11	10.77	11
QOCSPBO	11.53	12	11.80	12	11.80	12
SPBO	3.80	2	3.47	2	3.27	2
MESPBO	2.00	1	1.67	1	1.67	1

**Table 6:** The description of datasets used in the comparative study.

ID	Name	Number of Features	Number of Instances	Number of Classes
Dataset 1	Tic-Tac-Toe Endgame	90	958	2
Dataset 2	BreastCancer Wisconsin (Original)	10	699	2
Dataset 3	Statlog (Heart)	13	270	2
Dataset 4	Wine	13	178	3
Dataset 5	Congressional Voting Records	16	435	2
Dataset 6	Zoo	16	101	7
Dataset 7	Lymphography	18	148	4
Dataset 8	Hepatitis	19	155	2
Dataset 9	German Credit Dataset Analysis	20	1000	2
Dataset 10	Waveform	21	5000	3

**Table 7:** The results of MESPBO and other algorithms in fitness.

Function	Metric	PSO	SO	GRO	SBOA	ED	ESC	HHWOA	IGWO	MSPBO	QOCSPBO	SPBO	MESPBO
Dataset 1	Ave	0.1410	0.1435	0.1410	0.1535	0.1410	0.1443	0.1536	0.1410	0.1511	0.1410	0.1410	0.1410
	Std	3 × 10^−17^	0.0079	3 × 10^−17^	0.0079	3 × 10^−17^	0.0105	0.0133	3 × 10^−17^	0.0130	3 × 10^−17^	3 × 10^−17^	3 × 10^−17^
Dataset 2	Ave	0.0317	0.0317	0.0317	0.0317	0.0317	0.0317	0.0318	0.0317	0.0317	0.0317	0.0317	0.0317
	Std	3 × 10^−17^	3 × 10^−17^	3 × 10^−17^	3 × 10^−17^	3 × 10^−17^	3 × 10^−17^	0.0003	3 × 10^−17^	3 × 10^−17^	3 × 10^−17^	3 × 10^−17^	0
Dataset 3	Ave	0.1123	0.1123	0.1126	0.1123	0.1125	0.1123	0.1127	0.1123	0.1123	0.1123	0.1123	0.1122
	Std	1 × 10^−17^	1 × 10^−17^	1 × 10^−17^	0.003	1 × 10^−17^	1 × 10^−17^	0.005	1 × 10^−17^	1 × 10^−17^	1 × 10^−17^	1 × 10^−17^	1 × 10^−17^
Dataset 4	Ave	0.0015	0.0018	0.0015	0.0016	0.0015	0.0015	0.0017	0.0015	0.0015	0.0015	0.0015	0.0014
	Std	0	0.005	0	0.002	0	0	0.003	0	0	0	0	0
Dataset 5	Ave	0.0599	0.0612	0.0599	0.0572	0.0588	0.0513	0.0614	0.0471	0.0489	0.0476	0.0457	0.0441
	Std	1 × 10^−2^	1 × 10^−2^	1 × 10^−2^	1 × 10^−2^	1 × 10^−2^	1 × 10^−2^	1 × 10^−2^	1 × 10^−2^	1 × 10^−2^	1 × 10^−2^	9 × 10^−3^	6 × 10^−3^
Dataset 6	Ave	0.0012	0.0012	0.0013	0.0012	0.0012	0.0012	0.0013	0.0012	0.0012	0.0012	0.0012	0.00012
	Std	2 × 10^−4^	0	3 × 10^−4^	0	0	0	3 × 10^−4^	0	0	0	0	0
Dataset 7	Ave	1 × 10^−3^	2 × 10^−3^	2 × 10^−3^	2 × 10^−3^	2 × 10^−3^	1 × 10^−3^	1 × 10^−3^	2 × 10^−3^	2 × 10^−3^	2 × 10^−3^	2 × 10^−3^	2 × 10^−3^
	Std	8 × 10^−4^	1 × 10^−3^	7 × 10^−4^	9 × 10^−4^	3 × 10^−4^	8 × 10^−4^	7 × 10^−4^	6 × 10^−4^	8 × 10^−4^	9 × 10^−4^	8 × 10^−4^	0
Dataset 8	Ave	9 × 10^−2^	9 × 10^−2^	9 × 10^−2^	9 × 10^−2^	8 × 10^−2^	8 × 10^−2^	9 × 10^−2^	8 × 10^−2^	9 × 10^−2^	8 × 10^−2^	8 × 10^−2^	7 × 10^−2^
	Std	5 × 10^−2^	5 × 10^−2^	3 × 10^−2^	3 × 10^−2^	3 × 10^−2^	1 × 10^−2^	4 × 10^−2^	5 × 10^−2^	3 × 10^−2^	3 × 10^−2^	4 × 10^−4^	2 × 10^−4^
Dataset 9	Ave	0.2036	0.1939	0.1910	0.2125	0.1927	0.1981	0.2086	0.1911	0.1987	0.1940	0.1889	0.1861
	Std	0.0101	0.0111	0.0005	0.0128	0.0038	0.0077	0.0196	0.0081	0.0106	0.00048	0.0062	0.0084
Dataset 10	Ave	0.2036	0.1939	0.1910	0.2125	0.1927	0.1981	0.2086	0.1911	0.1987	0.1940	0.1889	0.1861
	Std	0.0101	0.0111	0.005	0.0128	0.0038	0.0077	0.0196	0.0081	0.0106	0.0048	0.0062	0.0084

**Table 8:** The results of MESPBO and other algorithms in accuracy.

Function	Metric	PSO	SO	GRO	SBOA	ED	ESC	HHWOA	IGWO	MSPBOO	QOCSPBOO	SPBO	MESPBO
Dataset 1	Ave	86.3%	86.1%	86.3%	86.1%	86.3%	86.0%	85.2%	86.3%	85.4%	86.3%	86.3%	86.1%
Dataset 2	Ave	97.1%	97.1%	97.1%	97.1%	97.1%	97.1%	97.1%	97.1%	97.1%	97.1%	97.1%	97.1%
Dataset 3	Ave	88.9%	88.9%	88.9%	88.9%	88.9%	88.9%	88.9%	88.9%	88.9%	88.9%	88.9%	89.0%
Dataset 4	Ave	100%	100%	100%	100%	100%	100%	100%	100%	100%	100%	100%	100%
Dataset 5	Ave	94.11%	93.95%	94.11%	94.57%	94.57%	95.04%	93.95%	94.11%	95.50%	94.37%	94.68%	95.81%
Dataset 6	Ave	100%	100%	100%	100%	100%	100%	100%	100%	100%	100%	100%	100%
Dataset 7	Ave	100%	100%	100%	100%	100%	100%	100%	100%	100%	100%	100%	100%
Dataset 8	Ave	91.11%	91.56%	91.11%	92.00%	92.00%	92.89%	88.44%	92.00%	92.68%	90.36%	91.27%	93.33%
Dataset 9	Ave	79.9%	80.7%	81.0%	79.0%	80.8%	80.3%	79.3%	81.0%	80.3%	80.7%	81.2%	81.5%
Dataset 10	Ave	79.9%	80.7%	81%	79%	80.8%	80.3%	79.3%	81%	80.3%	80.7%	81.2%	81.5%

**Table 9:** The results of MESPBO and other algorithms in average number of features.

Function	Metric	PSO	SO	GRO	SBOA	ED	ESC	HHWOA	IGWO	MSPBO	QOCSPBO	SPBO	MESPBO
Dataset 1	Ave	5	5.4	5	5.4	5	5.2	7	5	6.6	5	5	5
Dataset 2	Ave	3	3	3	3	3	3	3.1	3	3	3	3	3
Dataset 3	Ave	3	3	3	3.3	3	3	3.3	3	3	3	3	3
Dataset 4	Ave	2	2	2	2.1	2	2	2.2	2	2	2	2	2
Dataset 5	Ave	2.7	2.2	2.7	3.2	3.5	3.6	2.5	4.4	4.3	4.2	3.2	2.1
Dataset 6	Ave	2.12	2.2	2	2	2	2	2.2	2	2	2	2	2
Dataset 7	Ave	3.2	3.7	4.1	3.4	2.1	2.1	3.4	2.3	2	2	2	2
Dataset 8	Ave	3.2	3.7	4.1	3.4	2.2	2.1	3.5	2.3	2	2	2	2
Dataset 9	Ave	9.3	5.7	5.8	9.3	5.4	6.2	7.4	6	7.5	5.9	5.7	6
Dataset 10	Ave	9.3	5.7	5.8	9.3	5.4	6.2	7.4	6	7.5	5.9	6	5.7

**Table 10:** The ranges of unknown parameters.

Parameters	Single Diode PV Models	
	Lb	Ub
$I_{ph}\left(A\right)$	0	1
$I_{d}\left(\mu A\right)$	0	1
$R_{s}\left(\Omega \right)$	0	0.5
$R_{sh}\left(\Omega \right)$	0	100
n	1	2
$I_{d1}\left(\mu A\right)$	0	1
$I_{d2}\left(\mu A\right)$	0	1
$n_{1}$	1	2
$n_{1}$	1	2

**Table 11:** Comparison among different algorithms on SDM.

Algorithm	$I_{ph}\left(A\right)$	$I_{d}\left(\mu A\right)$	$R_{s}\left(\Omega \right)$	$R_{sh}\left(\Omega \right)$	n	RSME	sig
RTH	7.6043 × 10^−1^	9.9716 × 10^−7^	3.1402 × 10^−2^	1.1245 × 10^2^	1.6041 × 10^0^	9.8615 × 10^−4^	+
SAO	7.6084 × 10^−1^	1.0000 × 10^−6^	3.1385 × 10^−2^	1.0000 × 10^2^	1.6045 × 10^0^	9.8735 × 10^−4^	+
GRO	7.6070 × 10^−1^	3.3551 × 10^−7^	3.6241 × 10^−2^	5.5509 × 10^1^	1.4850 × 10^0^	9.8987 × 10^−4^	+
SO	7.6078 × 10^−1^	3.2067 × 10^−7^	3.6406 × 10^−2^	5.3452 × 10^1^	1.4805 × 10^0^	2.4000 × 10^−3^	+
ESC	7.6076 × 10^−1^	3.2096 × 10^−7^	3.6391 × 10^−2^	5.3553 × 10^1^	1.4805 × 10^0^	1.7000 × 10^−3^	+
INFO	7.6084 × 10^−1^	8.3538 × 10^−7^	3.2367 × 10^−2^	1.0000 × 10^2^	1.5834 × 10^0^	9.8654 × 10^−4^	+
SBOA	7.6078 × 10^−1^	3.2302 × 10^−7^	3.6377 × 10^−2^	5.3719 × 10^1^	1.4812 × 10^0^	9.8633 × 10^−4^	+
GKSO	7.6031 × 10^−1^	4.0577 × 10^−7^	3.5468 × 10^−2^	6.5852 × 10^1^	1.5045 × 10^0^	9.8678 × 10^−4^	+
IGWO	7.6061 × 10^−1^	3.6866 × 10^−7^	3.5871 × 10^−2^	5.8965 × 10^1^	1.4946 × 10^0^	1.1000 × 10^−3^	+
HHWOA	7.6107 × 10^−1^	2.1506 × 10^−7^	3.7916 × 10^−2^	4.1703 × 10^1^	1.4415 × 10^0^	9.8674 × 10^−4^	+
ED	7.6077 × 10^−1^	3.2394 × 10^−7^	3.6366 × 10^−2^	5.3675 × 10^1^	1.4815 × 10^0^	9.8870 × 10^−4^	+
MEED	7.6078 × 10^−1^	3.2305 × 10^−7^	3.6377 × 10^−2^	5.3721 × 10^1^	1.4812 × 10^0^	9.8602 × 10^−4^	/

**Table 12:** IAE of MESPBO on SDM.

MESPBO	$V\left(V\right)$	$I\left(A\right)$	$I_{sim}\left(A\right)$	${IAE}_{I}\left(A\right)$	$P_{sim}\left(W\right)$	${IAE}_{p}\left(A\right)$
1	−2.0570 × 10^−1^	7.6400 × 10^−1^	7.6205 × 10^−1^	1.9511 × 10^−3^	−1.5675 × 10^−1^	4.0134 × 10^−4^
2	−1.2910 × 10^−1^	7.6200 × 10^−1^	7.6137 × 10^−1^	6.3184 × 10^−4^	−9.8293 × 10^−2^	8.1570 × 10^−5^
3	−5.8800 × 10^−2^	7.6050 × 10^−1^	7.6074 × 10^−1^	2.4304 × 10^−4^	−4.4732 × 10^−2^	1.4291 × 10^−5^
4	5.7000 × 10^−3^	7.6050 × 10^−1^	7.6017 × 10^−1^	3.3215 × 10^−4^	4.3330 × 10^−3^	1.8932 × 10^−6^
5	6.4600 × 10^−2^	7.6000 × 10^−1^	7.5964 × 10^−1^	3.6187 × 10^−4^	4.9073 × 10^−2^	2.3377 × 10^−5^
6	1.1850 × 10^−1^	7.5900 × 10^−1^	7.5914 × 10^−1^	1.3832 × 10^−4^	8.9958 × 10^−2^	1.6391 × 10^−5^
7	1.6780 × 10^−1^	7.5700 × 10^−1^	7.5864 × 10^−1^	1.6371 × 10^−3^	1.2730 × 10^−1^	2.7470 × 10^−4^
8	2.1320 × 10^−1^	7.5700 × 10^−1^	7.5806 × 10^−1^	1.0560 × 10^−3^	1.6162 × 10^−1^	2.2513 × 10^−4^
9	2.5450 × 10^−1^	7.5550 × 10^−1^	7.5724 × 10^−1^	1.7442 × 10^−3^	1.9272 × 10^−1^	4.4391 × 10^−4^
10	2.9240 × 10^−1^	7.5400 × 10^−1^	7.5587 × 10^−1^	1.8746 × 10^−3^	2.2102 × 10^−1^	5.4813 × 10^−4^
11	3.2690 × 10^−1^	7.5050 × 10^−1^	7.5338 × 10^−1^	2.8778 × 10^−3^	2.4628 × 10^−1^	9.4077 × 10^−4^
12	3.5850 × 10^−1^	7.4650 × 10^−1^	7.4875 × 10^−1^	2.2510 × 10^−3^	2.6843 × 10^−1^	8.0698 × 10^−4^
13	3.8730 × 10^−1^	7.3850 × 10^−1^	7.4052 × 10^−1^	2.0165 × 10^−3^	2.8680 × 10^−1^	7.8098 × 10^−4^
14	4.1370 × 10^−1^	7.2800 × 10^−1^	7.2643 × 10^−1^	1.5667 × 10^−3^	3.0053 × 10^−1^	6.4814 × 10^−4^
15	4.3730 × 10^−1^	7.0650 × 10^−1^	7.0458 × 10^−1^	1.9228 × 10^−3^	3.0811 × 10^−1^	8.4082 × 10^−4^
16	4.5900 × 10^−1^	6.7550 × 10^−1^	6.7168 × 10^−1^	3.8212 × 10^−3^	3.0830 × 10^−1^	1.7539 × 10^−3^
17	4.7840 × 10^−1^	6.3200 × 10^−1^	6.2663 × 10^−1^	5.3700 × 10^−3^	2.9978 × 10^−1^	2.5690 × 10^−3^
18	4.9600 × 10^−1^	5.7300 × 10^−1^	5.6817 × 10^−1^	4.8281 × 10^−3^	2.8181 × 10^−1^	2.3948 × 10^−3^
19	5.1190 × 10^−1^	4.9900 × 10^−1^	4.9706 × 10^−1^	1.9389 × 10^−3^	2.5445 × 10^−1^	9.9254 × 10^−4^
20	5.2650 × 10^−1^	4.1300 × 10^−1^	4.1296 × 10^−1^	3.5430 × 10^−5^	2.1743 × 10^−1^	1.8654 × 10^−5^
21	5.3980 × 10^−1^	3.1650 × 10^−1^	3.1876 × 10^−1^	2.2600 × 10^−3^	1.7207 × 10^−1^	1.2200 × 10^−3^
22	5.5210 × 10^−1^	2.1200 × 10^−1^	2.1494 × 10^−1^	2.9383 × 10^−3^	1.1867 × 10^−1^	1.6222 × 10^−3^
23	5.6330 × 10^−1^	1.0350 × 10^−1^	1.0558 × 10^−1^	2.0816 × 10^−3^	5.9474 × 10^−2^	1.1726 × 10^−3^
24	5.7360 × 10^−1^	−1.0000 × 10^−2^	−6.5923 × 10^−3^	3.4077 × 10^−3^	−3.7813 × 10^−3^	1.9547 × 10^−3^
25	5.8330 × 10^−1^	−1.2300 × 10^−1^	−1.2585 × 10^−1^	2.8543 × 10^−3^	−7.3411 × 10^−2^	1.6649 × 10^−3^
26	5.9000 × 10^−1^	−2.1000 × 10^−1^	−2.1250 × 10^−1^	2.5047 × 10^−3^	−1.2538 × 10^−1^	1.4778 × 10^−3^

## Data Availability

The original contributions presented in this study are included in the article material. Further inquiries can be directed to the corresponding author.
